# Genotoxicity and Gene Expression in the Rat Lung Tissue following Instillation and Inhalation of Different Variants of Amorphous Silica Nanomaterials (aSiO_2_ NM)

**DOI:** 10.3390/nano11061502

**Published:** 2021-06-07

**Authors:** Fátima Brandão, Carla Costa, Maria João Bessa, Elise Dumortier, Florence Debacq-Chainiaux, Roland Hubaux, Michel Salmon, Julie Laloy, Miruna S. Stan, Anca Hermenean, Sami Gharbia, Anca Dinischiotu, Anne Bannuscher, Bryan Hellack, Andrea Haase, Sónia Fraga, João Paulo Teixeira

**Affiliations:** 1 EPIUnit—Instituto de Saúde Pública, Universidade do Porto, Rua das Taipas, 4050-600 Porto, Portugal; fatimabrandao.988@gmail.com (F.B.); cstcosta@gmail.com (C.C.); mjbessa8@gmail.com (M.J.B.); jpft12@gmail.com (J.P.T.); 2 Laboratory for Integrative and Translational Research in Population Health (ITR), 4050-600 Porto, Portugal; 3 Environmental Health Department, National Institute of Health Dr. Ricardo Jorge, Rua Alexandre Herculano 321, 4000-053 Porto, Portugal; 4 ICBAS—Institute of Biomedical Sciences Abel Salazar, U. Porto—University of Porto, Rua de Jorge Viterbo Ferreira 228, 4050-313 Porto, Portugal; 5 Unité de Recherche en Biologie Cellulaire (URBC), Namur Research Institute for Life Sciences (Narilis), University of Namur, 5000 Namur, Belgium; elise.dumortier@gmail.com (E.D.); florence.chainiaux@unamur.be (F.D.-C.); 6 StratiCELL Laboratories, Research and Development, 5032 Les Isnes, Belgium; rhubaux@straticell.com (R.H.); msalmon@straticell.com (M.S.); 7 Namur Nanosafety Centre, Department of Pharmacy, Namur Research Institute for Life Sciences (Narilis), University of Namur, 5000 Namur, Belgium; julie.laloy@unamur.be; 8 Department of Biochemistry and Molecular Biology, University of Bucharest, 050095 Bucharest, Romania; miruna.stan@bio.unibuc.ro (M.S.S.); anca.hermenean@gmail.com (A.H.); samithgh2@hotmail.com (S.G.); anca.dinischiotu@bio.unibuc.ro (A.D.); 9 “Aurel Ardelean” Institute of Life Sciences, “Vasile Goldis” Western University of Arad, 310414 Arad, Romania; 10 Department of Chemical and Product Safety, German Federal Institute for Risk Assessment (BfR), 10589 Berlin, Germany; anne.bannuscher@unifr.ch (A.B.); Andrea.Haase@bfr.bund.de (A.H.); 11 Adolphe Merkle Institute (AMI), University of Fribourg, 1700 Fribourg, Switzerland; 12 Institute of Energy and Environmental Technology (IUTA) e.V., 47229 Duisburg, Germany; bryan.hellack@uba.de; 13 German Environment Agency (UBA), 06844 Dessau-Roβlau, Germany

**Keywords:** silica nanomaterials, in vivo inhalation, in vivo instillation, rat lung, DNA damage, gene expression

## Abstract

Several reports on amorphous silica nanomaterial (aSiO_2_ NM) toxicity have been questioning their safety. Herein, we investigated the in vivo pulmonary toxicity of four variants of aSiO_2_ NM: SiO_2__15_Unmod, SiO_2__15_Amino, SiO_2__7 and SiO_2__40. We focused on alterations in lung DNA and protein integrity, and gene expression following single intratracheal instillation in rats. Additionally, a short-term inhalation study (STIS) was carried out for SiO_2__7, using TiO_2__NM105 as a benchmark NM. In the instillation study, a significant but slight increase in oxidative DNA damage in rats exposed to the highest instilled dose (0.36 mg/rat) of SiO_2__15_Amino was observed in the recovery (R) group. Exposure to SiO_2__7 or SiO_2__40 markedly increased oxidative DNA lesions in rat lung cells of the exposure (E) group at every tested dose. This damage seems to be repaired, since no changes compared to controls were observed in the R groups. In STIS, a significant increase in DNA strand breaks of the lung cells exposed to 0.5 mg/m^3^ of SiO_2__7 or 50 mg/m^3^ of TiO_2__NM105 was observed in both groups. The detected gene expression changes suggest that oxidative stress and/or inflammation pathways are likely implicated in the induction of (oxidative) DNA damage. Overall, all tested aSiO_2_ NM were not associated with marked in vivo toxicity following instillation or STIS. The genotoxicity findings for SiO_2__7 from instillation and STIS are concordant; however, changes in STIS animals were more permanent/difficult to revert.

## 1. Introduction

Silica (SiO_2_) nanomaterials (NM) are currently produced in large-scale and are amongst the most widely commercialized manufactured NM. SiO_2_ NM are easily synthesized in different size, shape, crystallinity, porosity, and surface chemistry enabling their use in different fields from biomedicine to cosmetics, in the food industry or for bioremediation [1,2,3]. More specifically, amorphous silica nanomaterials (aSiO_2_ NM) are being extensively used for industrial purposes as biosensors and catalytic supports, stabilizers of emulsions or foams for enhanced oil recovery processes, for the improvement of the mechanical characteristics of polymers and composites, or as additives for paints/lacquers/coatings [1,3]. This wide range of industrial applications and growing commercial production obviously increased the likelihood of human exposure to aSiO_2_ NM, mainly through inhalation [4,5].

While crystalline SiO_2_ has been classified as carcinogenic to humans (Group 1) by the International Agency for Research on Cancer (IARC) [6], aSiO_2_ has not been listed as hazardous to humans by the Organization for Economic Co-operation and Development (OECD) and European Centre for Ecotoxicology and Toxicology of Chemicals (ECETOC) [7,8] neither as carcinogenic by IARC. In the nanoparticulate form, the toxicological potential of the synthetic amorphous silica (SAS) has been questioned in the last years by several reports on in vitro and in vivo toxicity as reviewed in Murugadoss et al. [5]. Increasing evidence support the ability of NM to cause genotoxicity through direct or indirect mechanisms [9,10,11,12,13,14]. Depending on their size, NM may reach the nucleus passing through the nuclear pores or during cell division when the nuclear envelope disassembles, directly interacting with DNA [15,16,17,18]. However, reports on genetic damage induced for most NM are consistent with indirect genotoxic effects rather than direct interaction of NM with DNA, encompassing chromosomal aberrations, micronuclei formation, mutations, DNA adducts and DNA strand breaks and oxidative damage [14,19,20,21,22]. Genotoxicity caused by NM interactions with proteins involved in DNA repair or in antioxidant response has also been reported [23]. Oxidative stress is one of the most reported triggering factors for NM-induced genotoxicity. Increasing evidence shows that elicitation of oxidative stress mechanisms by NM may contribute to pro-inflammatory disease processes in the lung [24]. Along with DNA, ROS can cause oxidative damage in other cellular biomolecules such as proteins and lipids. Proteins are major targets for oxidative modification, predominantly protein carbonyl formation, as they can scavenge up to 70% of the generated ROS [25,26]. Carbonylation of proteins is an irreversible modification that often leads to a decrease or loss of protein function [27].

So far, pulmonary toxicity of aSiO_2_ NM remains poorly understood. While some in vivo studies reported oxidative stress and reversible inflammation accompanied by granuloma formation or fibrogenesis [13,28,29,30,31,32,33,34,35], others showed no signs of toxicity following exposure to these NM [36,37]. Regarding the genotoxic potential of aSiO_2_ NM in the lung tissue, from the available in vivo studies addressing this issue in rodents [4,13,36,38,39], only one of them revealed significant genotoxicity [39]. In that study, Nemmar et al. [39] observed oxidative stress, inflammation and DNA damage in all organs studied (lung, heart, liver, kidney, and brain) at 24 h after a single intraperitoneal administration (0.25 mg/kg) of 50 nm aSiO_2_ NM in mice and proposed that oxidative stress and/or inflammation were most likely linked to the induced DNA damage. However, most of the existing studies reported an inflammatory response after exposure to aSiO_2_ NM but no changes in DNA integrity [4,13].

The available in vitro studies on the genotoxic potential of aSiO_2_ NM in human or rodent pulmonary cells are also scarce [9,10,11,13,40]. Mu et al. [40] observed an induction of DNA damage following exposure to 10 μg/mL of 14 nm aSiO_2_ NM in three different cell lines, colonic (HT-29), skin (HaCat) and lung (A549) cells. In turn, Maser et al. [13] reported a size-dependent increase in DNA strand breaks in Chinese hamster lung fibroblast V79 cells. These authors observed an increase in DNA strand breaks in V79 cells exposed to 15 nm aSiO_2_ NM but not in cells exposed to 55 nm aSiO_2_ NM. Indeed, size seems to play a role for NM genotoxicity potential. Gonzalez et al. [9] investigated the effects of differently sized aSiO_2_ NM (12, 28, 40, 59, 139 and 174 nm) in micronucleus (MN) induction and cell cycle changes in human alveolar epithelial A549 cells under serum and serum-free conditions. These authors observed MN induction at lower concentrations with larger aSiO_2_ NM being more active under serum-free conditions. Furthermore, they also detected cell cycle changes induced by four aSiO_2_ NM (40, 59, 139 and 174 nm) out of the six aSiO_2_ NM tested in the absence but not in the presence of serum in the incubation medium, showing that serum levels of the incubation medium strongly influence NM toxicity. Additionally, Decan et al. [10] also observed a size-dependent increase in MN frequency in mouse lung epithelial (FE1) cells exposed to 12 nm, 5–10 nm, and 10–15 nm aSiO_2_ NM.

From the studies described above, it is evident that there is a lack of correlation between in vivo and in vitro findings concerning the genotoxicity of aSiO_2_ NM. In contrast with the absence of significant genotoxic effects in vivo, most of the in vitro studies revealed genotoxic responses from exposure to aSiO_2_ NM. Consequently, no definitive conclusion on the pulmonary effects of aSiO_2_ NM and on the mechanisms involved in such responses can be drawn from the available evidence.

The comet assay is an indicator test for detection of DNA damage that is recommended by the Organization for Economic Co-operation and Development (OECD) to assess genotoxicity in vivo [41]. This assay has proved to be a sensitive and relatively simple method to study specific DNA lesions such as single and double-strand breaks, oxidation and alkylation lesions or crosslinks, and so far, it is one the most used assay in nanogenotoxicology [42,43]. Moreover, by incorporating a step of digestion of DNA with lesion-specific endonucleases (e.g., formamidopyrimidine DNA glycosylase (FPG) to recognize oxidized purines), makes it possible to measure oxidized bases and to monitor oxidative stress [44].

Considering the knowledge gap on the in vivo genotoxic potential of aSiO_2_ NM, we carried out a study to understand the role of the physicochemical properties (size and surface chemistry) of aSiO_2_ NM in pulmonary toxicity, on lung DNA and protein integrity, and gene expression. So far, only a few studies have investigated the genotoxicity and gene expression changes in rodent models following exposure to NM. In this regard, Li et al. [45] reported that intraperitoneal injection of 10 nm anatase TiO_2_ NP provoked oxidative DNA lesions associated with oxidative stress, inflammatory responses and apoptosis in the lung tissue. On the other hand, perturbation of the expression of genes involved in key cellular processes, such as DNA damage and repair, accompanied by increased levels of primary and oxidative DNA damage, were found in the lung tissue of mice intravenously injected with silver (Ag) and TiO_2_ NP [46]. In turn, Gosens et al. [47] showed that pathways related to inflammation and cell proliferation were significantly activated in rats after short-term inhalation with copper oxide (CuO) NM.

In the first phase of our study, effects of in vivo exposure to four aSiO_2_ variants (i.e., differently capped—unmodified and amino-modified; differently sized—7, 15 and 40 nm), were investigated in a rat model following intratracheal instillation. These four aSiO_2_ NM variants were chosen based on previous in vitro studies that described changes in the proteome and metabolome of rat alveolar epithelial RLE-6TN cells in response to these aSiO_2_ NM [48]. Since inhalation often better represents the natural route to aerosols and particulate matter exposure, a short-term inhalation study (STIS) was performed using SiO_2__7 NM and TiO_2_-NM105 as a benchmark to verify in vivo instillation findings. TiO_2_ NM-105 was used as a benchmark material since it has been used in many other case studies [12,49,50,51,52], thus allowing the comparison of data derived from different studies. Primary and oxidative DNA damage, protein carbonylation and alterations in gene expression, with a focus on inflammation and oxidative stress related genes, were evaluated in the lung tissue following exposure and after a recovery period.

## 2. Materials and Methods

### 2.1. Chemicals

All chemicals used were of high purity or analytical grade. Triton X-100, low melting point (LMP) agarose, Tris hydrochloride (Tris-HCl) and methyl methanesulfonate (MMS) were purchased from Sigma-Aldrich (Madrid, Spain). Dimethyl sulfoxide (DMSO), sodium hydroxide (NaOH), sodium chloride (NaCl), potassium chloride (KCl) and potassium hydroxide (KOH) were bought from Merck KGaA (Darmstadt, Germany). Tris base and disodium salt dihydrate (Na_2_EDTA) were purchased from Merck Millipore (Madrid, Spain). Phosphate buffered saline (PBS) was purchased from Lonza (Lutterworth, UK). Normal melting point (NMP) agarose was supplied by Bioline (London, UK). Formamidopyrimidine DNA glycosylase (FPG) was bought from New England, Biolabs (Ipswich, MA, USA). SYBR^®^ Gold was purchased from Thermo Fisher Scientific (Madrid, Spain).

### 2.2. Nanomaterials (NM)

In the present study, four aSiO_2_ NM variants differing in structure, size and surface charge, were tested: SiO_2__15_Unmod and SiO_2__15_Amino (capped with aminopropyltrimethoxysilane) provided by the BASF SE (Mannheim, Germany), and SiO_2__7 and SiO_2__40 provided by Evonik Industries Resource Efficiency GmbH (Kirschenallee, Germany). Rutile-anatase titanium nanoparticles (TiO_2__NM105) obtained from the JRC repository served as a benchmark. The tested NM were sterilized by gamma-irradiation, extensively characterized using state-of-the-art techniques and confirmed to be endotoxin-free by the Limulus amebocyte lysate (LAL) test [50]. In Table S1 are presented some of their main physicochemical properties, namely surface capping, zeta potential, redox potential, and dissolution rate [50,53].

### 2.3. In Vivo Studies

#### 2.3.1. Intratracheal Instillation Study

**Figure 1:**
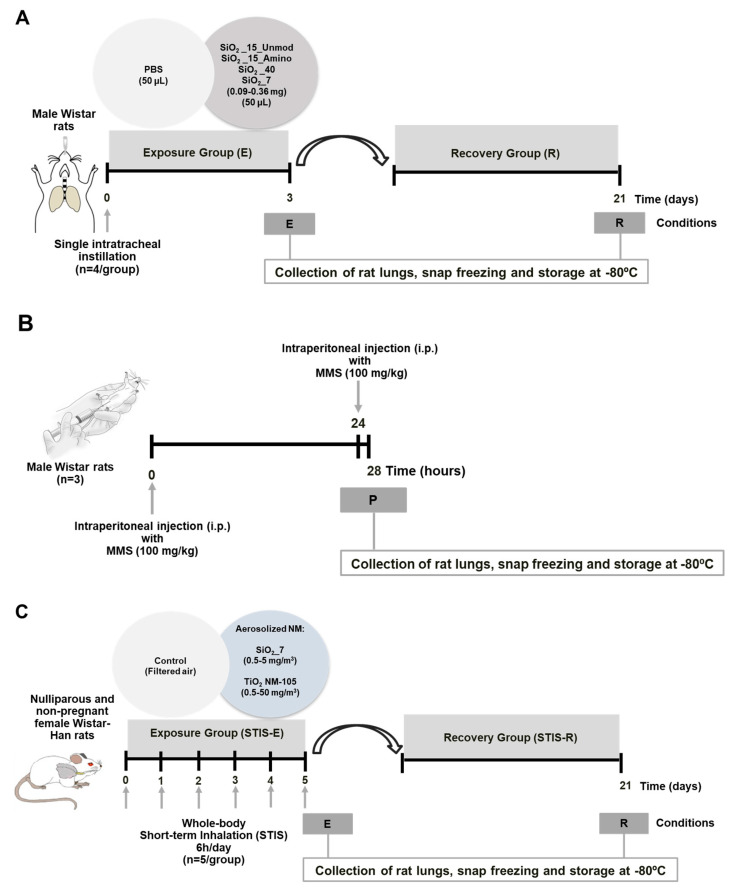
Experimental design of the in vivo studies. (**A**) Male Wistar rats were exposed by intratracheal installation to different doses (0.09, 0.18, 0.36 mg/animal; *n* = 4/group) of four aSiO_2_ NM variants (SiO_2__15_Unmod, SiO_2__15_Amino, SiO_2__40 and SiO_2__7) freshly dispersed (Cup-Horn, NanoToxClass SOP) in 50 µL sterile PBS. The control group was intratracheally instilled with the same amount of sterile PBS (Ct; *n* = 4). The animals were euthanized either 3 days (Exposure group—E) or 21 days (Recovery group—R) after exposure. (**B**) Male Sprague Dawley rats intraperitoneally (i.p.) injected with methyl methanesulfonate (MMS) at a dose of 100 mg/kg (*n* = 3), 28 and 4 h before animal euthanasia served as positive controls for the genotoxicity studies. (**C**) Short-term inhalation studies (STIS) were performed in nulliparous and non-pregnant female Wistar-Han rats (*n* = 5/group). The animals were exposed (whole-body) during 5 consecutive days for 6 h/day to the aerosolized SiO_2__7 (0.5, 2, 5 mg/m^3^) and TiO_2__NM105 (0.5, 2, 10, 50 mg/m^3^). Control groups (Ct) were exposed to filtered air. The rats were euthanized at 5 days (Exposure group—STIS-E) after the initial exposure or after a recovery period of 21 days (Recovery group—STIS-R).

The study was approved by the Ethics Committee of the “Vasile Goldis” Western University of Arad (Romania) and authorized by the Romanian National Sanitary Veterinary and Food Safety Authority by registration No. 007/27.11.2017. Male Wistar rats were maintained in acclimatization and quarantine for 10 days upon arrival. Then, the animals were housed in pathogen-free individually ventilated cages at 20–25 °C, 40–60% relative humidity, 12-h light/dark cycle and had free access to food and water. The experimental design is depicted in Figure 1A. The study has been conducted under the framework of ERA-NET SIINN NanoToxClass project, as previously described by Bannuscher et al. [54]. Briefly, aSiO_2_ NM (SiO_2__15_Unmod, SiO_2__15_Amino, SiO_2__40 and SiO_2__7) were dispersed by indirect probe sonication using a Bandelin Cup Horn (Berlin, Germany) according to the internal Standard Operating Procedure (SOP) of NanoToxClass consortium [55]. Briefly, rats were anesthetized by an intraperitoneal (i.p.) injection of a ketamine/xylazine cocktail (100/10 mg/kg body weight) and were intratracheally instilled with different doses (0.09, 0.18 or 0.36 mg per animal) of freshly dispersed SiO_2__15_Unmod, SiO_2__15_Amino, SiO_2__7 or SiO_2__40 in 50 µL of sterile PBS (*n* = 4 rats/dose/time of exposure). The control animals (*n* = 4 rats/group) were instilled with the same volume of sterile PBS. The rats were euthanized under anesthesia at 3 days (exposure group, E) or 21 days (recovery group, R) after instillation. The lungs were perfused with saline solution (0.9% NaCl), collected, snap frozen in liquid nitrogen and stored at −80 °C until analysis. Positive controls for the genotoxicity study were constituted by rats (*n* = 3) that received i.p. methyl methanesulfonate (MMS), two doses of 100 mg/kg body weight with a difference of 24 h, the animals being sacrificed 4 h after from the second administration (Figure 1B).

#### 2.3.2. Short-Term Inhalation Study (STIS)

STIS has been also conducted under the framework of NanoToxClass project, as previously described by Bannuscher et al. [54]. The study was approved by the Committee on the Ethics of Animal Experiments of the University of Namur, Belgium under the agreement UN 18306 DO. Nulliparous and non-pregnant female Wistar-Han rats (Charles River, France) with age of 7–8 weeks were housed in pathogen-free individually ventilated cages (Type GR900, Tecniplast, Dison, Belgium) at 20–22 °C, 40–65% relative humidity, 12-h light/dark cycle and had free access to food and water. Whole-body exposure to the aerosols was conducted as previously described by Lozano et al. [56]. The aerosols were generated by an RBG-1000 (Palas GmbH, Karlsruhe, Germany) generator and analyzed in real-time by ELPI analyzer (Dekati Ltd., Kangasala, Finland), capable of particle measurement between 7 nm and 10 µm [56]. SiO_2__7 has been selected for this study based on OMICs data analyses that revealed more pronounced effects at pulmonary level in the rats instilled with this variant [54]. Selection of the tested doses was based on the calculation of the deposited doses used in the in vivo instillation study using the Multiple-Path Particle Deposition model (MPPD, version 3.04, ARA) to assure prevention of overload effects. Thus, rats (*n* = 5/dose) were exposed to aerosolized SiO_2__7 (0.5, 2 and 5 mg/m^3^), TiO_2__NM105 (0.5, 2, 10 and 50 mg/m^3^) or to filtered air (controls) during 6 h/day for 5 consecutive days. Effective doses were calculated and are presented in Table 1. Stress, morbidity, and mortality were monitored at 30 min, 1 h, and 18 h after exposure. Rats were euthanized by i.p. injection of 60 mg/kg of pentobarbital (Nembutal, Ceva Sante Animale, France) at two time points: immediately after the final exposure (exposure groups, E) or after a recovery period of 21 days (recovery groups, R), as presented in Figure 1C. Euthanasia was followed by a macroscopic exam and an autopsy. Lungs were perfused with saline solution, collected, snap frozen using liquid nitrogen and stored at −80 °C until analysis.

### 2.4. Comet Assay

#### 2.4.1. Lung Cells Isolation for Comet Assay

Cell isolation from the lung tissue was carried out according to Li et al. (2017) and Pfuhler et al. (2017), with slight modifications. Briefly, the lung tissue (0.5–2 g) was placed in an ice-cold Petri dish, washed, and then immersed in 2 mL of ice-cold tissue mincing buffer (Hank’s Balanced Salt Solution with 20 mM EDTA and 10% DMSO, pH 7.5). The tissue was gently minced with a scalpel and the resulting cell suspension filtered through a 70 μm nylon Corning^®^ (Corning, NY, USA) cell strainer in order to separate the individual cells from the remaining macerated tissues, which were transferred to a microcentrifuge tube and further homogenized with a pestle in 1 mL of ice-cold tissue mincing buffer. The resulting cell suspension was also filtered through the 70 µm cell strainer. Total cell suspension was centrifuged for 5 min at 120× *g* and the obtained pellet resuspended in 3 mL of ice-cold mincing buffer. Aliquots of the cell suspensions were slowly frozen and stored at −80 °C until analysis.

#### 2.4.2. Alkaline and Formamidopyrimidine DNA Glycosylase (FPG)-Modified Comet Assay

The alkaline comet assay was performed as previously described by Bessa et al. [57] with slight modifications. Minimum Information for Reporting Comet Assay procedures and results (MIRCA) recommendations were followed in this manuscript [58]. Briefly, cell suspensions were thawed on ice and then centrifuged for 5 min at 500× *g*. The resulting pellets were resuspended in PBS pH 7.4 and cell density determined using a Neubauer cell chamber. After cell counting, 1.6 × 10^4^ cells/200 µL in PBS pH 7.4 were transferred to a microtube and centrifuged for 5 min at 500× *g*. Subsequently, the supernatants were discarded, and cells embedded in 200 µL of 1% LMP agarose. Five microliters of each cell suspension (400 cells) were placed onto dry microscope slides precoated with 1% NMP using a medium throughput 12-gel comet assay unit (Severn Biotech Ltd.^®^, Kidderminster, UK). After agarose solidification at 4 °C for 5 min, slides were immersed in ice-cold lysis solution (2.5 M NaCl, 100 mM Na_2_EDTA, 10 mM Tris-base, 10 M NaOH pH 10, 1% Triton-X 100) during 1 h at 4 °C, protected from light. After lysis, FPG-modified comet assay slides were washed three times for 5 min with buffer F (0.1 M KCl, 0.5 mM Na_2_EDTA, 40 mM HEPES, 0.2 mg/mL BSA, pH 8) prior incubation for 30 min at 37 °C with 2.7 U/mL FPG enzyme or with buffer F alone (negative control). The alkaline comet assay slides were washed 3 times with PBS pH 7.4 for 5 min. After incubation, FPG and Buffer F slides were washed with PBS pH 7.4. For DNA unwinding, all slides were immersed in the electrophoresis platform for 40 min in freshly prepared electrophoresis solution (1 mM Na_2_EDTA, 0.3 M NaOH, pH 13) followed by electrophoresis in the same solution for 30 min at constant 25 V (0.9 V/cm) and 400 mA. At the end of electrophoresis, the slides were washed with ice-cold PBS pH 7.4 and deionized water for 10 min each, followed by fixation with 70% ethanol and 96% ethanol for 10 min each, and the slides dried overnight. Prior to scoring, slides were stained with a 1:10,000 dilution of SYBR^®^ Gold in Tris-EDTA buffer (10 mM Tris-HCl, 1 mM EDTA, pH 7.5–8). The slides were scored using a fluorescence microscope (Motic BA410 ELITE, Barcelona, Spain) attached to an epifluorescence illuminator (Filter Set: Exciter D480/30×, Emitter D535/40 nm, Dichroic 505DCLP) with 100× magnification, using the image analysis software Comet Assay IV (Perceptive Instruments, Suffolk, UK). At least 100 cells/experimental group (50 in each replicate gel) were analyzed in each slide. Percentage of DNA in the comet tail (% tail intensity; TDNA) was used as DNA damage descriptor. Net FPG-sensitive sites were calculated by subtracting the % TDNA value of Buffer F slide from the score of the FPG slide.

### 2.5. Protein Carbonylation Analysis of the Rat Lung Tissue

Protein carbonylation was quantified similar to the procedure described in Bahl et al. [59]. Deep frozen lung tissues were homogenized in a cryomill. Total protein lysates were prepared using a modified RIPA buffer. Protein concentrations were measured using a Bradford assay according to manufacturer instructions. Protein carbonyls were assessed using the OxyBlot^®^ kit (Merck Millipore, Darmstadt, Germany), which uses the derivatization reagent 2,4-dinitrophenylhydrazine (DNPH). DNPH reacts with the carbonyl groups and is transformed to 2,4-dinitrophenylhydrazones which can be detected with specific antibodies. Protein carbonyls were assessed using a Dot Blot technique, which permits to assess the signal of protein carbonylation and to normalize it to the total deposited protein determined by Colloidal Gold Total Protein Stain (Bio-Rad, Hercules, CA, USA). The results are expressed as fold increase in comparison to lysates obtained from untreated, control animals (i.e., controls). Semi-quantification relative to controls (signal from untreated, control animals) was performed using Image Lab^TM^ software (Bio-Rad, Hercules, CA, USA) with a global background subtraction. For each NM, at least three independent biological replicates were measured.

### 2.6. Gene Expression

#### 2.6.1. RNA Extraction and Purification

Deep-frozen lungs were homogenized via cryomill (1.7 amplitude, 2–8 min, Analysette 3, Fritsch GmbH, Idar-Oberstein, Germany) with continuous usage of liquid nitrogen. For total RNA extraction 25 mg of the pulverized lungs were used. To enable proper storage conditions, RNA extraction was immediately started using the RNeasy^®^ kit (Qiagen, Hilden, Germany) after lung homogenization, according to the manufacturer’s recommendations. Briefly, lysis buffer was added to the frozen lung powder and the mixture was transferred to QIAshredder^TM^ (Qiagen, Hilden, Germany). The obtained flowthroughs were stored at −80 °C until further work-off, according to manufacturer’s recommendations. Samples were purified from remaining DNA with on-column digestion using RNase-Free DNase Set (Qiagen, Hilden, Germany). The RNA concentration was determined by spectrophotometric measurement (QIAxpert, Qiagen, Hilden, Germany) and the RNA quality was analyzed by capillarity electrophoresis (Agilent Bioanalyzer 2100-Agilent RNA 6000 Nano Kit, Cat No. 5067-1511, Agilent Scientific Instruments, Santa Clara, CA, USA). The integrity of the total RNA was assessed by visualization of intact ribosomal RNA bands.

#### 2.6.2. Gene Expression Analysis of the Rat Lung Tissue—Affymetrix Analysis

The amplification of total RNA and conversion into cDNA was performed in 3 steps on 50 ng with the Ribo-SPIA technology (Ovation Pico WTA System V2, NuGEN, TECAN, Leek, The Netherlands) and amplified samples were purified using the Agencourt RNA Clean up XP Beads, Cat No. A29168 (Agencourt-Beckman Coulter Genomics, Suarlée, Belgium). For each amplified cDNA, fragmentation, and biotin labelling was processed on 5 μg with the NuGEN Encore Biotin Module Cat No. 4200-12 (NuGEN, TECAN, Leek, The Netherlands). Quality control of amplified product before and after fragmentation and labelling was analyzed on the Agilent Bioanalyzer. Hybridization, washing, and staining were performed according to the Affymetrix user manual. The hybridization cocktail was prepared using the Affymetrix GeneChip^®^ Expression Amplification Reagent Hybridization Controls (Cat No. 900454) and the Hybridization Module for GeneChip^®^ Hybridization, Wash and Stain Kit (Cat No. 900720) and mixed with amplified cDNA samples. GeneChip^®^ Rat Clariom S Arrays (Cat No. 902921) were treated according to the manual instruction and prepared for hybridization. Hybridization was performed overnight for 18 h in the GeneChip^®^ Hybridization Oven 640. Washing and staining of the arrays was done in the GeneChip^®^ Fluidics Station 450 and scanning with GeneChip^®^ Scanner 3000 (Affymetrix, Santa Clara, CA, USA). The processing of the raw data was carried out with software R (v3.4.3; [60]) and the “oligo” package (v1.42.0; [61]) of the Bioconductor project (v3.2; [62]).

#### 2.6.3. Gene Expression Analysis of the Rat Lung Tissue—Custom TaqMan Array Cards (TAC)

Custom TaqMan Array (TAC) 384-well microfluidic cards (ThermoFisher Scientific, Waltham, MA, USA) were used to detect 48 different transcript species, including 4 housekeeping genes (*18s RNA, ACTB, GAPDH, PPIA*) previously tested as stable in our conditions and 44 genes of interest associated with inflammation and stress response (Table S2). Briefly, total RNA (1 µg) was reverse transcribed using the “High-Capacity cDNA Reverse Transcription” kit (ThermoFisher Scientific, Waltham, MA, USA) following manufacturer’s instructions. Each TAC fill reservoir was loaded with PCR reaction mix containing 100 ng of cDNA and 1x TaqMan Universal Master Mix II, no Uracil-DNA Glycosylase (UNG) (ThermoFisher Scientific, Waltham, MA, USA). TAC was centrifuged for 1 min at 1200 rpm in a Heraus Multifuge 3S centrifuge (Cardinal Health, Dublin, OH, USA) and then sealed. The PCR amplifications were performed in a ViiA7 thermal cycler (ThermoFisher Scientific, Waltham, MA, USA): 2 min at 95 °C, followed by 40 cycles of denaturation at 95 °C for 15 s and annealing/extension at 60 °C for 1 min. mRNA abundances values were normalized to the expression value of *18s RNA, ACTB, GAPDH, PPIA* and calculations based on the comparative C_t_ (cycle threshold) method (2^−ΔΔCt^) as described by Schmittgen and Livak [63] using the Viia7 version 1.2 software (ThermoFisher Scientific, Waltham, MA, USA). Following amplification, data from 8 transcript species could not be used because they were expressed at too low level (C_t_ > 30) or not detected (*A2M, Bdkrb1, HRH1, HRH2, IFNg, IL6, IL10, PTGS1*). In total, the expression of 36 genes of interest were, therefore, analyzed.

### 2.7. Statistical Analysis

Statistical analyses of comet assay results were performed using GraphPad Prism 6 for Windows (La Jolla, CA, USA). Comet assay results are expressed as a mean ± standard deviation (SD). Data were tested for normality and homogeneity of variances by Shapiro–Wilk and Levene’s tests, respectively. When the distribution of the response variables followed a normal distribution, a two-way analysis of variance (ANOVA) followed by post hoc Dunnett’s test for multiple comparisons was performed to test the differences in % tail intensity data between control and exposed rats within the exposure or recovery groups. Nonparametric tests were applied when data distribution significantly deviated from normality; the Kruskal–Wallis test was applied for comparison between groups and Dunn’s multiple comparisons test was performed to test the differences in % tail intensity data between control and exposed rats within the exposure or recovery groups. The individual statistical analyses of genes/transcripts from the Affymetrix analyses were performed with the “Moderated t” method implemented in the R Limma 3.26.8. Gene set analysis of the Affymetrix data was performed using mean log *p*-value (MLP). In turn, TAC results were presented as the fold change relative to the respective control conditions ± SD and differences in gene expression analyzed with Student’s *t*-test. The probability level of 0.05 was used as the criterion of significance.

## 3. Results

### 3.1. Primary and Oxidative DNA Damage in the Rat Lung Tissue

**Figure 2:**
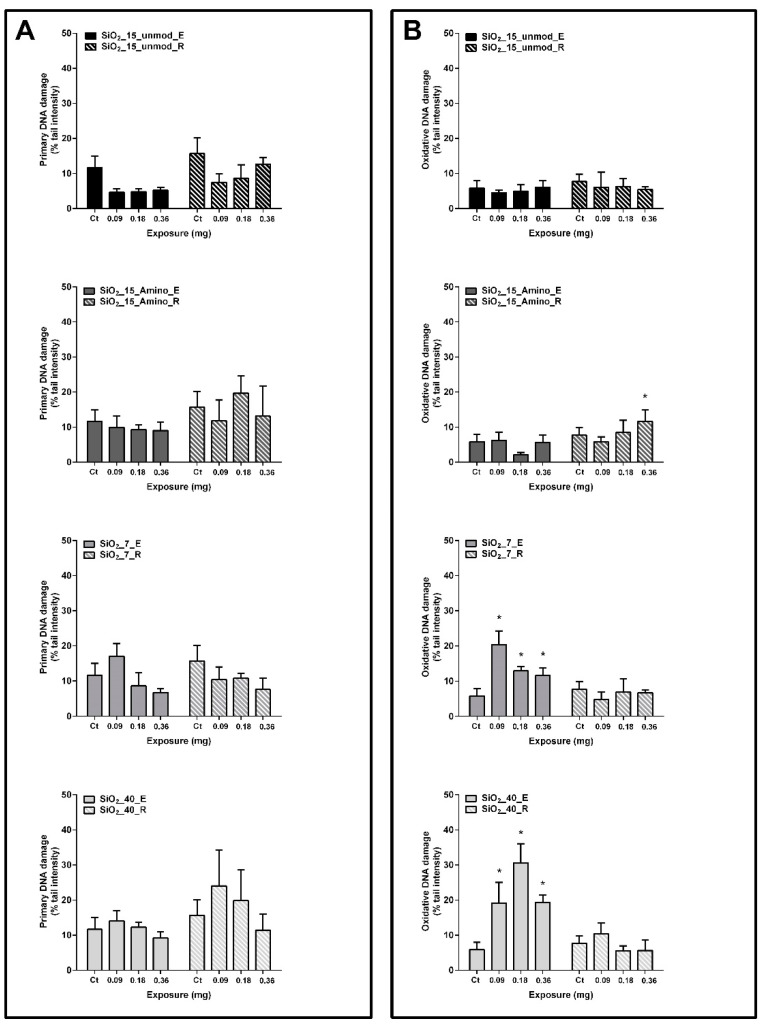
Primary (**A**) and oxidative (**B**) DNA damage in the rat lung tissue after 3 days (exposure group; E) and 21 days (recovery group; R) of a single intratracheal instillation (0.09–36 mg) of different variants of aSiO_2_ NM (SiO_2__15_Unmod, SiO_2__15_Amino, SiO_2__7 and SiO_2__40), as assessed by the comet assay. Percentage of DNA in the tail (% tail intensity) was used as DNA damage descriptor. Columns represent mean and error bars are the standard deviation (SD); Ct (negative control). * *p* < 0.05 vs. Ct.

Primary and oxidative DNA damage were evaluated in total lung cell suspensions by the standard and FPG-modified alkaline comet assay versions, respectively. In the instillation study, DNA damage was assessed in animals at 3 days (Exposure group; E) and 21 days (Recovery group; R) after initial instillation of the different aSiO_2_ NM variants (SiO_2__15_Unmod, SiO_2__15_Amino, SiO_2__7 and SiO_2__40). The obtained results are presented in Figure 2. DNA strand breaks of lung cells of rats exposed to all tested aSiO_2_ NM variants, at any tested dose, did not differ from the controls (Figure 2A). However, some changes in the level of the FPG-sensitive sites have been detected following exposure to SiO_2__15_Amino, SiO_2__7 and SiO_2__40. Thus, a significant increase in % tail intensity value of lung cells from rats instilled with the highest dose of SiO_2__15_Amino (0.36 mg) compared to the respective control group has been detected (11.64 ± 3.27 vs. 7.68 ± 2.12) in the recovery (SiO_2__15_Amino_R; at 21 days after NM instillation) but not in the exposure group (SiO_2__15_Amino_E; at 3 days after NM instillation). In contrast, oxidative DNA damage in the lung tissue of animals exposed to SiO_2__7 and SiO_2__40 was significantly increased in the exposure but not in the recovery groups. Therefore, exposure to all doses of SiO_2__7 (SiO_2__7_E) caused a significant induction of FPG-sensitive sites, being the highest % tail intensity value observed for the lowest tested dose (0.09 mg; 20.33 ± 3.93% tail intensity). Rats exposed to all tested doses of SiO_2__40 also exhibited a significant increase in the FPG-sensitive sites compared to the control group (Figure 2B), being the highest value observed for the intermediate dose tested (0.18 mg; 30.56 ± 5.49% tail intensity).

**Figure 3:**
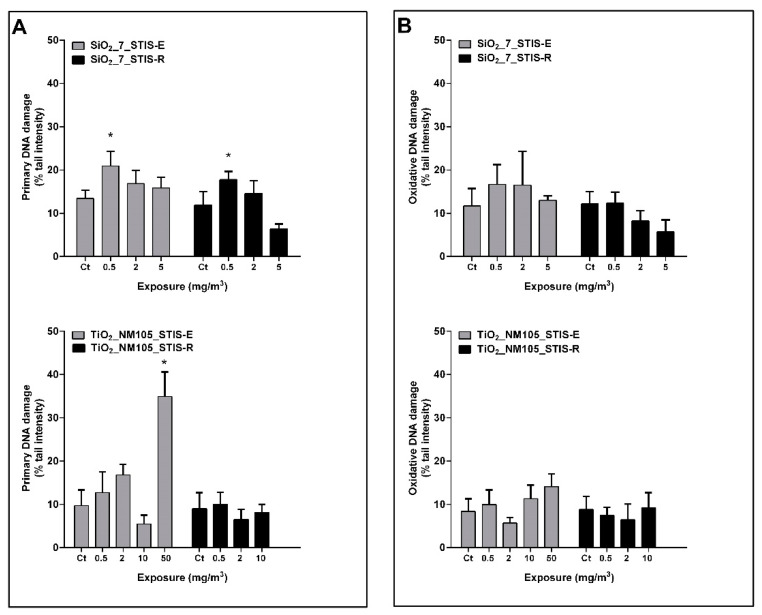
Primary (**A**) and oxidative (**B**) DNA damage in the rat lung tissue following short-term inhalation exposure (STIS) to aerosolized SiO_2_ _7 (0.5–5 mg/m^3^) and TiO_2_ _NM105 (0.5–50 mg/m^3^), assessed by the comet assay at 5 days after the initial exposure (exposure group; STIS-E) and after a recovery period of 21 days (recovery group; STIS-R). Percentage of DNA in the tail (% tail intensity) was used as DNA damage descriptor. Columns represent mean and error bars are the standard deviation (SD); Ct (negative control). * *p* < 0.05 vs. Ct.

In the inhalation study, rats have been exposed to SiO_2__7 and TiO_2__NM105. The lowest tested dose of aerosolized SiO_2__7 (0.5 mg/m^3^) caused a significant increase in primary DNA strand breaks both in the STIS-E (20.91 ± 3.42 vs. 13.42 ± 1.86% tail intensity) and STIS-R (17.72 ± 1.94 vs. 11.82 ± 3.18% tail intensity) compared to the respective controls (Figure 3A). Exposure to doses of TiO_2__NM105 up to 10 mg/m^3^ did not induce significant changes in DNA damage in the lung tissue in the STIS-E and STIS-R groups (Figure 3A). However, lung cells of rats exposed to 50 mg/m^3^ TiO_2__NM105 exhibited a significant increase in DNA strand breaks comparing with control (34.91 ± 5.66 vs. 9.68 ± 3.65% tail intensity) (Figure 3A). Moreover, exposure to both types of aerosolized NM did not cause a significant increase in lung oxidative DNA damage in both groups (Figure 3B). As expected, DNA damage in the lung of MMS-injected animals showed a significant increase in the % tail intensity (74.50 ± 4.28) compared with the control group (data not shown).

### 3.2. Protein Carbonylation in the Rat Lung Tissue

Protein carbonylation, which has been found as a good marker for oxidative stress [53], was evaluated in the rat lung tissue of the exposure groups (E) from both instillation and STIS. Data was expressed as fold changes relative to control and categorized according to the scoring system indicated in Table 2, established based on expert judgement. For overall categorization of each group, the highest score was considered. The results for the instillation study are depicted in Table 3. As shown, according to the level of protein carbonyls detected in rat lung tissues, SiO_2__15_Unmod, SiO_2__7 and SiO_2__40 presented a low oxidative stress potential, while SiO_2__15_Amino revealed a medium oxidative stress potential. On the other hand, SiO_2__7 was classified as having medium oxidative stress potential, whereas TiO_2__NM105 had a high oxidative stress potential (Table 4).

### 3.3. Gene Expression

#### 3.3.1. Gene Expression Alterations in the Rat Lung Tissue—Affymetrix Analysis

In order to gain further knowledge of the global cell response and pathways involved at the whole transcriptome level, we performed an Affymetrix analysis on the RNA samples available from animals exposed to the highest tested dose of SiO_2__15_Unmod and SiO_2__15_Amino (0.36 mg), both at 3 days (Exposure group; E) and 21 days (Recovery group; R) after initial instillation. These two NM were selected for this exploratory transcriptomic screening based on previous in vitro and in vivo evidence of cytotoxicity and changes in metabolome and proteome profile of oxidative stress-related pathways [53]. Thus, a gene group analysis using the Gene Ontology Biological Process [64] revealed that the most affected pathways in animals exposed to SiO_2__15_Unmod were related to microtubule dynamics, cytoskeleton, chromosomal replication, as well as inflammation. The recovery group showed similar responses with alterations of the microtubules, DNA repair, and glucocorticoids pathways. Similar results were detected in animals exposed to SiO_2__15_Amino regarding the microtubule dynamics, with involvement of the glucocorticoid response (cortisol) already in the exposure group (3 days), while for rats exposed to SiO_2__15_Unmod and SiO_2__15_Amino, a stronger immune response has been observed in the recovery group (21 days) (Table 5). Of note, SiO_2__15___Unmod_E and SiO_2__15_Amino_E shared 27 out of the 100 most significant gene expression changes (Table 6), including an increase in osteopontin (secreted phosphoprotein 1; spp1) of 4- and 2.7-fold, respectively. Both groups also showed a strong induction of the gene coding for the matrix metallopeptidase 12 (3.6- and 4.3-fold, respectively). In the most significant genes shared in both recovery groups, we observed a decrease in expression of genes coding for structural proteins involved in tissue cohesion, such as Epithelial membrane protein 1 (*Emp1*, 0.5- and 0.3-fold, respectively), and Collagen type VI alpha 1 (*Col6a1*, 0.7- and 0.5-fold, respectively).

#### 3.3.2. Gene Expression Alterations in the Rat Lung Tissue—TAC Analysis

Inflammation and oxidative stress are two common hallmarks of NM exposure that can lead to potential induction of DNA damage [42,43,65]. Based on Affymetrix’s analyzed data, we decided to explore the expression of several genes involved in inflammation and stress response following in vivo NM exposure using a Custom TaqMan Array 384-well microfluidic Cards (TAC). This technique allows the simultaneous screening of the relative expression of selected transcripts in several conditions [66]. Among 48 different cDNA species analyzed, including 44 genes of interest associated with inflammation and stress response, 40 were successfully amplified by TAC including four reliable housekeeping genes (*18s RNA, ACTB, GAPDH, PPIA*) and 36 genes of interest. TaqMan analysis was performed in the lung tissue of animals intratracheally instilled with the highest tested dose (0.36 mg) of SiO_2__7 and SiO_2__40 and in animals exposed by STIS to all tested doses (0.5 mg/m^3^, 2 mg/m^3^ and 5 mg/m^3^) of SiO_2__ 7, both from the exposure (E) and recovery (R) groups.

For in vivo instillation studies, there has been relatively little change in the abundance of transcripts species whatever the NM (SiO_2__7 or SiO_2__40), and whatever the assessed time-point, i.e., at 3 days (E group) or 21 days (R group) after exposure (Table 7). In the lung tissue of SiO_2__7-exposed animals, two genes were found upregulated (*HMOX-1* and *Plcd1*) in the E group and two genes were upregulated (*Casp3* and *Vcam1*) in the R group. Despite of the low number, the significant alterations were known to be important markers for biological processes such as oxidative stress (*HMOX-1*, *Plcd1*), immune response (*VCAM-1*) and apoptosis (*Casp-3*). Similar results were observed upon SiO_2__40 treatment with one gene (*SOD2*) in E group and two genes (*IL-1b* and *Il1rl1*) in R group significantly enhanced. Even though the number of significantly regulated genes to be limited, these are also connected to oxidative stress (*SOD2*) and inflammatory responses (*IL-1b* and *Il1rl1*).

In STIS animals exposed to the aerosolized SiO_2__7, many changes in lung gene expression have been detected compared to control animals, although far more were present in STIS_E than in the STIS_R group (Table 8). Those changes were dose dependent in both groups, ranging from six altered genes at 0.5 mg/m^3^, 12 at 2 mg/m^3^ to 19 at 5 mg/m^3^, where downregulated (e.g., *Adrb2*) as upregulated (e.g., *IL-1b*) genes have been detected. The alterations in gene expression detected for all tested doses in the STIS_E group are linked to increased inflammation (*TNF*, *IL-1b*), as well as oxidative stress (*Nos2*) and beta-adrenergic signaling (*Adrb2*). At the highest dose, also signs of high level of inflammation and oxidative stress became apparent due to the expression of *CXCL1* (neutrophil chemotaxis), *TNF*, *Itgam*, *Nos2* and *SOD2*. In the STIS_R group, changes on gene expression were less pronounced, only occurring gene downregulation compared to the controls, which seems to indicate a recovery to the control condition.

When comparing the impact on lung gene expression of exposure to SiO_2__7, markedly more significant changes occurred upon STIS than upon instillation. Though, it should be kept in mind that this was a very limited transcriptomic approach with just 40 genes investigated. However, important hints into biological processes involved in NM response in vivo were gained.

## 4. Discussion

Herein, we have investigated the pulmonary effects, i.e., putative genotoxicity, protein carbonylation and alterations in the expression of related genes in rats exposed to aSiO_2_ NM either by instillation or inhalation. Inhalation exposure enables a continuous low deposition and retention rate of the test material along all the respiratory tract better mimicking exposure to airborne particulates, while instillation bypasses the upper respiratory tract and delivers an abrupt and concentrated dose to the lungs, which results in highly uneven patterns of lung retention [67,68].

Most of the in vivo instillation and inhalation studies for aSiO_2_ NM available in the literature report negative outcomes in terms of genotoxicity [4,13,36,65]. Guichard et al. [4] showed that three consecutive intratracheal instillations of SiO_2_ NP (NM-200, NM-201, NM-202, and NM-203) at doses of 3, 6, or 12 mg/kg did not induce any significant primary and oxidative DNA damage in the rat lung. Maser et al. [13] also demonstrated that after a single intratracheal instillation of 360 μg, neither 15 nm SiO_2_ NPs nor 55 nm SiO_2_ NP caused genotoxic effects in the rat lung. In addition, Sayes et al. [36] reported that one or three days of exposure to SiO_2_ NP (37 and 83 nm; 1.8 or 86 mg/m^3^) aerosols did not induce any genotoxic effect in the rat lung. In the present study, intratracheal instillation of all tested doses of the aerosolized SiO_2__7 and SiO_2__40 caused an increase in oxidative DNA lesions in the exposure groups. Interestingly, neither SiO_2__7 nor SiO_2__40-exposed rats exhibited altered expression of 8-oxoguanine DNA glycosylase (*OGG1*), a gene encoding for an oxidative DNA repair enzyme. Notwithstanding, DNA damage seems to be repaired since no differences were seen in the recovery groups comparing with the respective controls. In turn, exposure to any tested dose of SiO_2__15_Unmod did not cause significant changes in DNA integrity, while SiO_2__15_Amino induced a slight, but significant increase in the oxidative DNA damage in the lung cells of the recovery group exposed to the highest tested dose (0.36 mg). These results are in good agreement with oxidative stress potential of these NM based on protein carbonyl levels of the lung cells from the exposure group, where SiO_2__15_Amino (classified as medium potential) caused higher protein carbonylation than SiO_2__15_Unmod (classified as low potential). Thus, amino surface capping seems to endow SiO_2__15_Amino of greater reactivity. Notwithstanding, this finding contrast with previous in vivo reports demonstrating that surface capping with amino groups attenuates the toxicity of the SiO_2_ NM [30,69]. These differences may be explained by variations in studies experimental design such as characteristics of the animals used (species and sex) and/or the assessed endpoints. Furthermore, we cannot discard the possibility of SiO_2__15_Amino have partially or even totally lost their surface amino groups in vivo.

Pathway analysis of the lung tissue of rats intratracheally instilled with 0.36 mg of SiO_2__15_Unmod or SiO_2__15_Amino also revealed some gene expression alterations that may help to understand their effects upon DNA integrity. Rat lung is a complex organ composed by several types of cells including endothelial cells, interstitial cells, alveolar type II cells, alveolar type I cells, and alveolar macrophages. In the alveoli, the particles can be taken up by alveolar macrophages, which are critical in inflammation initiation, maintenance, and resolution [51,70,71,72]. Indeed, inflammation that is generally triggered to recruit cells from the systemic circulation to aid in the resolution of the lung insult [73], was one of the most affected biological pathways in the exposure group (SiO_2__15_Unmod_E). On the other hand, glucocorticoid secretion-related pathways, which are involved in immunosuppressive and anti-inflammatory responses [74,75,76] have also been affected in the recovery group (SiO_2__15_Unmod_R). These findings seem to indicate that regulatory inflammation mechanisms have been set in motion to deal with the activation of inflammatory pathways observed in SiO_2__15_Unmod_E animals. In turn, pathways related to cortisol were found to be affected in the SiO_2__15_Amino exposure (SiO_2__15_Amino_E) but not in the recovery (SiO_2__15_Amino_R) group.

Changes in the expression of genes associated with cytoskeleton and microtubule pathways have also been detected. In the exposure groups (SiO_2__15_Unmod_E and SiO_2__15_Amino_E), upregulation of genes coding for proteins involved in extracellular matrix (ECM) and tissue remodeling (*Retpla*, *Spp1* and *Mmp12*) have been observed. For instance, osteopontin (secreted phosphoprotein 1; *Spp1*) has been associated with the regulation of inflammation and mucin production in a murine model of asbestosis (85). Thus, these alterations are likely to be associated with the activation of innate immune defenses to protect the respiratory tract from the instilled NM through the production of mucus by epithelial cells [77] and epithelial pulmonary tissue repair that involves cell migration allowing the formation of a temporary barrier, which is characterized by down-regulation of tight junctions and increased expression of matrix metalloproteinases and ECM components [78]. Supporting this view, are also alterations in the expression of genes coding for structural proteins involved in tissue cohesion (e.g., *Emp1* and *Col6a*) observed in both recovery groups (SiO_2__15_Unmod_R and SiO_2__15_Amino_R) that most likely contribute for cell rearrangement for restoring normal tissue architecture [78,79].

TEM analysis of the intracellular localization of NM can greatly facilitate and provide useful information for understanding of the biological effects of NM [80,81,82]. Overall, NM localized in the mitochondria, lysosomes or within the nucleus are considered more hazardous than the ones localized in the cytoplasm [83,84,85,86]. Increased levels of phosphatidylcholine (PC) metabolite PCaaC42:1 in the rat lung of the SiO_2__40_E group has been reported [54]. PC is one the most abundant and important glycerophospholipid for the structure of plasma and nuclear membranes [87], as such changes in its levels might have affected membranes fluidity thereby facilitating NM entrance into the cell, its direct contact with the nucleus, favoring the occurrence of genotoxic events.

Acellular ROS generated by SiO_2_ NM have also been shown to be an important contributor to its toxicity [88]. In this regard, NM physicochemical characterization can provide important clues. Measurements of redox potential, i.e., NM affinity for electrons and its ability to form ROS by Electron Paramagnetic Resonance (EPR) spectroscopy showed that either SiO_2__7 and SiO_2__40 are endowed with some intrinsic reactivity (Table S1; [50]). Low levels of protein carbonylation in the lung tissue of SiO_2__7 and SiO_2__40-exposed animals have been detected, which would suggest a low oxidative stress potential for both NM. Nevertheless, significant gene expression alterations in inflammation and oxidative stress related pathways have been observed following instillation of SiO_2__7 (*HMOX-1* and *Plcd1*) and SiO_2__40 (*SOD2*), supporting that oxidative DNA damage in rat lung caused by exposure to these two NM may arise from activation of cellular mechanisms in response to oxidative stress. Metabolomic analysis of the lung tissue obtained from in vivo instillation and STIS animals revealed alterations in the levels of several oxidative stress related metabolites such as methionine sulfoxide (Met-SO), hydroxy-proline (hydroxy-Pro) and spermidine concordant with a status of oxidative stress [54]. However, at 21 days after initial instillation, increased levels of metabolites such spermidine, which is a potent antioxidant, have been detected in the rat lung tissue of the SiO_2__15_Unmod, SiO_2__15_Amino and SiO_2__40-exposed animals that most likely contributed to counteract the oxidative stress in the recovery groups and mitigate NM-induced oxidative DNA damage.

Regarding DNA damage analysis of the lung tissue of STIS rats exposed to the aerosolized SiO_2__7, data revealed a significant increase in primary DNA damage at the lowest tested dose (0.5 mg/m^3^) either in rat lung cells of the exposure or recovery groups. Data on gene expression showed upregulation of genes encoding proteins related to inflammation processes (*I-1b*, *TNF* and *Nos2*) and oxidative stress (*Nos2*) suggesting that DNA damage in the lung may arise from activation of inflammatory mechanisms and oxidative stress. This possibility is supported by the fact that lung tissue of SiO_2__7_E rats exposed to the lowest dose exhibited increased levels of long-chain acylcarnitines (C18:2, C16:2-OH, C16:1 and C18), accompanied by reduced levels of phosphatidylcholine (Pcaa C36:3 and PCae C36:4) and lysophosphatidylcholine (LysoPCa 18:2) [51]. Indeed, increased levels of long-chain acylcarnitines levels are strongly associated with mitochondrial fatty acid oxidation (FAO) dysregulation and mitochondrial homeostasis disruption [89,90], whereas decreased levels of phosphatidylcholine molecules longer than 36 carbons are indicative of poor membrane condition [91].

Previous studies have shown that STIS are well suited for NM hazard ranking and for predicting NM effects that occur upon sub-chronic or chronic exposure [28,30,92,93]. Overall, data on DNA damage analysis of the lung tissues of STIS rats exposed to the aerosolized SiO_2__7 are in line with those found in the in vivo instillation study. Nevertheless, contrasting with the observed in the instillation study, DNA damage in the animals exposed to aerosolized SiO_2__7 was not repaired even though the reduction on gene expression was less pronounced in the recovery group, which seems to indicate a return to control levels. In STIS, TiO_2__NM105 was used as a benchmark NM. So far, no concordant genotoxic profile has been established for TiO_2_ NM, with the route of exposure and dose influencing the genotoxic outcome [94,95]. Nevertheless, several in vivo inhalation and instillation studies showed negative genotoxicity outcomes for TiO_2_ NM. Naya et al. [96] reported that single (1.0 or 5.0 mg/kg body weight) or repeated intratracheal instillation (0.2 or 1.0 mg/kg body weight; five weeks/once a week) of TiO_2_ anatase NP induced an inflammatory response, but not DNA damage as assessed by comet assay, in lung tissues of rats. Lindberg et al. [97] showed that inhalation of the highest dose of TiO_2_ NP (anatase) aerosols (28.2 mg/m^3^) induced an inflammatory response in mice BAL fluid not associated with significant genotoxic effects in lung epithelial cells. At the same time, Relier et al. [98] found that only under overload conditions (three instillations of 10 mg/kg) TiO_2__NM105 (rutile-anatase) induced delayed genotoxicity in lung, associated with persistent inflammation. In the present study, only rats exposed for five consecutive days to the highest dose of TiO_2__NM105 aerosols exhibited an increase in the DNA damage of total lung cells, which is consistent with its high oxidative stress potential based on the high levels of protein carbonylation found in the lung tissue of the exposed animals.

Inflammation and genotoxicity have also been reported following instillation and/or inhalation of other types of NM. In this regard, Jacobsen et al. [99] showed that quantum dots (QD) had greatest effects followed by carbon black (CB) and single-walled carbon nanotubes (SWCNT), with fullerene C60 and gold nanoparticles (AuNP) being least inflammatory and DNA-damaging in instilled mice. For their similarity with asbestos fibers, carbon nanotubes have been widely investigated for lung toxicity. Their toxicological potential is strongly dependent on physicochemical features such as nominal length and diameter, with long and rigid carbon nanotubes inducing persistent inflammation [100,101].

## 5. Conclusions

Amorphous silica nanomaterials (aSiO_2_ NM) are being extensively used in several industrial branches and are already available in the market in many consumer products. In our study, we have investigated effects on DNA integrity, protein carbonylation and gene expression in the lung tissue of rats exposed to different variants of aSiO_2_ NM, either by instillation or STIS (whole-body). Under our experimental conditions, aSiO_2_ NM did not induce marked in vivo toxicity, though some punctual changes have been detected. Overall, pyrogenic aSiO_2_ NM (SiO_2__7 and SiO_2__40) caused more pronounced effects on the assessed endpoints compared to colloidal aSiO_2_ NM (SiO_2__15_Unmod and SiO_2__15_Amino). Though similar findings were observed for the NM administered either way (SiO_2__7), its effects on DNA integrity and gene expression were more permanent and/or difficult to reverse in STIS than after instillation, which is likely to be explained by the different pattern of NM deposition along the respiratory tree following STIS and instillation, i.e., a more generalized vs a more localized distribution of NM, respectively. DNA damage caused by exposure to aSiO_2_ NM was found to be associated with changes in cellular architecture, inflammation, and oxidative stress pathways but not to alterations in DNA repair capacity, at least the one mediated by OGG1. Nevertheless, the detected changes did not follow a clear dose-dependent trend as were mostly observed at the highest tested dose. In summary, the obtained insights are important for unravelling aSiO_2_ NM toxicity mechanisms and future studies should be carried out for clarifying in vivo dose–response relationship for aSiO_2_ NM.

## Figures and Tables

**Table 1:** Comparison of in vivo applied and calculated effective doses of SiO_2__7 and TiO_2__NM105 in STIS.

NM	Exposure Conditions	Applied Dose [mg/m^3^]	Effective Dose [mg/m^3^]
SiO_2__7	Exposure group	0.5	0.74
		2	2.58
		5	5.02
	Recovery group	0.5	0.97
		2	1.41
		5	5.30
TiO_2__NM105	Exposure group	0.5	2.5
		2	4.92
		10	14.98
		50	41.97
	Recovery group	0.5	2.47
		2	3.41
		10	11.57

**Table 2:** Oxidative stress potential scoring based on protein carbonylation.

Protein Carbonylation (Relative to Controls, %)	Category	Oxidative Stress Potential
100 ± 15	1	None (No)
115–150	2	Low (L)
150–200	3	Medium (M)
>200	4	High (H)

**Table 3:** Protein carbonylation (values are fold changes relative to control) in rat lung following NM instillation.

NM	0.09 mg	0.18 mg	0.36 mg	Overall Category
SiO_2__15_Unmod_E	0.80 (No)	1.30 (L)	0.71 (No)	L
SiO_2__15_Amino_E	1.09 (No)	0.76 (No)	1.67 (M)	M
SiO_2__7_E	1.03 (No)	0.51 (No)	1.30 (L)	L
SiO_2__40_E	1.16 (L)	0.78 (No)	0.74 (No)	L

**Table 4:** Protein carbonylation (values are fold changes relative to control) in rat lung following NM inhalation.

NM	0.5 mg/m^3^	2 mg/m^3^	5 mg/m^3^	10 mg/m^3^	50 mg/m^3^	Overall Category
SiO_2__7 STIS-E	0.79 (L)	1.01 (L)	1.57 (M)	NA	NA	M
TiO_2__NM105_STIS-E	0.68 (L)	1.04 (L)	NA	1.41 (M)	5.53 (H)	H

**Table 5:** Main affected pathways in Gene Ontology (GO) in rats exposed by instillation to SiO_2__15_Unmod and SiO_2__15_Amino.

NM	Exposure Condition	Rank	Id	Size	*p* Value	Description
**SiO_2__15_Unmod**	**Exposure Group**	1	GO:0007127	83 (112)	6.00 × 10^−7^	meiosis I
		2	GO:0048247	42 (46)	3.50 × 10^−6^	lymphocyte chemotaxis
		3	GO:0071459	15 (21)	5.20 × 10^−6^	protein localization to chromosome, centromeric
		4	GO:0060340	10 (11)	8.20 × 10^−6^	positive regulation of type I interferon-mediated
		5	GO:0070098	63 (66)	1.30 × 10^−5^	chemokine-mediated signaling pathway
		6	GO:0072676	73 (78)	2.00 × 10^−5^	lymphocyte migration
		7	GO:0045132	67 (93)	3.20 × 10^−5^	meiotic chromosome segregation
		8	GO:0034501	13 (18)	4.20 × 10^−5^	protein localization to kinetochore
		9	GO:0034502	64 (76)	1.80 × 10^−4^	protein localization to chromosome
		10	GO:0070192	53 (72)	1.90 × 10^−4^	chromosome organization involved in meiotic cell cycle
	**Recovery Group**	1	GO:0048680	9 (11)	2.20 × 10^−5^	positive regulation of axon regeneration
		2	GO:0070572	10 (12)	8.80 × 10^−5^	positive regulation of neuron projection
		3	GO:2001223	10 (11)	1.60 × 10^−4^	negative regulation of neuron migration
		4	GO:2000849	11 (11)	1.60 × 10^−4^	regulation of glucocorticoid secretion
		5	GO:0071300	65 (76)	3.30 × 10^−4^	cellular response to retinoic acid
		6	GO:0003348	6 (6)	3.60 × 10^−4^	cardiac endothelial cell differentiation
		7	GO:0060956	6 (6)	3.60 × 10^−4^	endocardial cell differentiation
		8	GO:0006297	5 (5)	3.70 × 10^−4^	nucleotide-excision repair, DNA gap filling
		9	GO:0035933	12 (12)	5.10 × 10^−4^	glucocorticoid secretion
		10	GO:2000852	5 (5)	5.50 × 10^−4^	regulation of corticosterone secretion
**SiO_2__15_Amino**	**Exposure Group**	1	GO:0000910	96 (110)	1.50 × 10^−7^	cytokinesis
		2	GO:0000281	42 (47)	4.00 × 10^−7^	mitotic cytokinesis
		3	GO:0033260	23 (30)	5.40 × 10^−6^	nuclear DNA replication
		4	GO:0071459	15 (21)	7.60 × 10^−6^	protein localization to chromosome, centromeric
		5	GO:0051383	12 (14)	1.80 × 10^−5^	kinetochore organization
		6	GO:0051414	7 (7)	2.00 × 10^−5^	response to cortisol
		7	GO:0040020	31 (35)	2.30 × 10^−5^	regulation of meiotic nuclear division
		8	GO:0061640	49 (56)	2.50 × 10^−5^	cytoskeleton-dependent cytokinesis
		9	GO:0051445	45 (51)	2.50 × 10^−5^	regulation of meiotic cell cycle
		10	GO:1902969	9 (10)	3.90 × 10^−5^	mitotic DNA replication
	**Recovery Group**	1	GO:0035082	44 (60)	1.90 × 10^−14^	axoneme assembly
		2	GO:0003341	44 (64)	7.10 × 10^−13^	cilium movement
		3	GO:0001578	66 (89)	4.80 × 10^−8^	microtubule bundle formation
		4	GO:0002385	25 (39)	2.60 × 10^−5^	mucosal immune response
		5	GO:0060294	15 (17)	3.50 × 10^−5^	cilium movement involved in cell motility
		6	GO:0030199	38 (44)	5.10 × 10^−5^	collagen fibril organization
		7	GO:0001539	19 (22)	5.20 × 10^−5^	cilium or flagellum-dependent cell motility
		8	GO:0060285	19 (22)	5.20 × 10^−5^	cilium-dependent cell motility
		9	GO:0002251	26 (40)	5.20 × 10^−5^	organ or tissue specific immune response
		10	GO:0048755	13 (13)	6.50 × 10^−5^	branching morphogenesis of a nerve

Rats were exposed by instillation to 0.36 mg of SiO_2__15_Unmod or SiO_2__15_Amino and euthanized to isolate total RNA from the lungs both at 3 days (Exposure group; E) and 21 days (Recovery group; R) after initial instillation.

**Table 6:** Lung gene expression changes shared by rats exposed by instillation to SiO_2__15_Unmod and SiO_2__15_Amino.

Gene Symbol	Gene Name	3 Days_E			
		SiO_2__15_Unmod		SiO_2__15_Amino	
		*p* Value	FC	*p* Value	FC
*Retnla*	resistin like alpha	1.61 × 10^−5^	4.37	1.40 × 10^−6^	6.00
*Spp1*	secreted phosphoprotein 1	2.00 × 10^−5^	4.05	7.86 × 10^−4^	2.67
*Slc26a4*	solute carrier family 26-member 4	2.72 × 10^−5^	5.77	3.57 × 10^−4^	3.95
*Clvs2*	clavesin 2	4.04 × 10^−5^	0.60	2.74 × 10^−4^	0.66
*Zbp1*	Z-DNA binding protein 1	5.22 × 10^−5^	0.49	1.55 × 10^−3^	0.60
*Nupr1*	nuclear protein 1, transcriptional regulator	1.47 × 10^−4^	1.64	1.18 × 10^−3^	1.48
*Gas2l3*	growth arrest-specific 2 like 3	1.51 × 10^−4^	2.01	2.23 × 10^−4^	1.96
*Cfi*	complement factor I	1.78 × 10^−4^	2.22	8.85 × 10^−4^	1.96
*Ect2*	epithelial cell transforming 2	2.52 × 10^−4^	2.53	4.20 × 10^−5^	3.00
*Qrfpr*	pyroglutamylated RFamide peptide receptor	3.43 × 10^−4^	2.39	8.19 × 10^−4^	2.21
*Cdk1*	cyclin-dependent kinase 1	4.72 × 10^−4^	2.32	5.76 × 10^−5^	2.81
*Clybl*	citrate lyase beta like	5.64 × 10^−4^	1.90	3.12 × 10^−5^	2.33
*LOC100909700*	CD177 antigen-like	5.97 × 10^−4^	2.55	1.88 × 10^−5^	3.68
*Lcn2*	lipocalin 2	6.94 × 10^−4^	2.32	1.71 × 10^−4^	2.64
*Hmmr*	hyaluronan-mediated motility receptor	8.12 × 10^−4^	2.25	1.30 × 10^−4^	2.67
*Fbxo5*	F-box protein 5	8.13 × 10^−4^	1.81	2.49 × 10^−4^	1.96
*Cdkn3*	cyclin-dependent kinase inhibitor 3	1.07 × 10^−3^	2.36	1.85 × 10^−4^	2.82
*Olr196*	olfactory receptor 196	2.04 × 10^−3^	0.69	6.58 × 10^−4^	0.66
*Mmp12*	matrix metallopeptidase 12	2.09 × 10^−3^	3.65	7.07 × 10^−4^	4.36
*Wdr35*	WD repeat domain 35	2.09 × 10^−3^	1.59	1.46 × 10^−3^	1.62
*Ndc80*	NDC80 kinetochore complex component	2.15 × 10^−3^	2.19	7.57 × 10^−4^	2.42
*Cxcl9*	C-X-C motif chemokine ligand 9	2.17 × 10^−3^	1.84	6.51 × 10^−4^	2.02
*Nusap1*	nucleolar and spindle associated protein 1	2.26 × 10^−3^	2.89	1.49 × 10^−3^	3.06
*Ncapg*	non-SMC condensin I complex, subunit G	2.39 × 10^−3^	1.99	5.72 × 10^−4^	2.26
*L2hgdh*	L-2-hydroxyglutarate dehydrogenase	3.08 × 10^−3^	1.42	6.31 × 10^−4^	1.53
*Cenpw*	centromere protein W	3.33 × 10^−3^	2.20	3.75 × 10^−4^	2.77
*Fabp5*	fatty acid binding protein 5	3.45 × 10^−3^	1.43	1.56 × 10^−4^	1.66
**Gene symbol**	**Gene name**	**21 days_R**			
		**SiO_2__15_Unmod**		**SiO_2__15_Amino**	
		***p*** **value**	**FC**	***p*** **value**	**FC**
*Nrg1*	neuregulin 1	2.55 × 10^−5^	2.05	1.71 × 10^−4^	2.35
*Olfml3*	olfactomedin-like 3	3.8 × 10^−4^	0.66	8.39 × 10^−6^	0.43
*Gas8*	growth arrest specific 8	4.69 × 10^−4^	1.47	5.29 × 10^−5^	1.96
*Emp1*	epithelial membrane protein 1	6.32 × 10^−4^	0.53	1.41 × 10^−4^	0.35
*Col6a1*	collagen, type VI, alpha 1	1.13 × 10−^3^	0.69	6.35 × 10^−5^	0.49
*Tsnaxip1*	translin-associated factor X interacting protein 1	1.76 × 10^−3^	1.62	2.92 × 10^−5^	2.82
*Cfap45*	cilia and flagella associated protein 45	2.45 × 10^−3^	1.87	4.48 × 10^−4^	2.95
*Creg1*	cellular repressor of E1A-stimulated genes 1	2.92 × 10^−3^	1.50	1.05 × 10^−4^	2.30
*Syt5*	synaptotagmin 5	3.41 × 10^−3^	1.58	1.62 × 10^−4^	2.51
*Pigr*	polymeric immunoglobulin receptor	3.94 × 10^−3^	1.48	3.09 × 10^−4^	2.12
*Tekt4*	tektin 4	3.96 × 10^−3^	1.44	6.31 × 10^−5^	2.25

Rats were exposed by instillation to 0.36 mg of SiO_2__15_Unmod or SiO_2__15_Amino and euthanized to isolate total RNA from the lungs either at 3 days (Exposure group; E) and 21 days (Recovery group; R) after initial instillation.

**Table 7:** Gene expression profile identified by TAC analysis following instillation of SiO_2__7 or SiO_2__40 in rats.

		SiO_2__7		SiO_2__40	
Gene Symbol	NM	3 Days_E	21 Days_R	3 Days_E	21 Days_R
*Adrb2*	NM_012492	0.96 ± 0.10	1.06 ± 0.22	0.78 ± 0.02	0.94 ± 0.19
*Anxa3*	NM_012823	1.26 ± 0.35	0.93 ± 0.08	0.60 ± 0.04 *	0.98 ± 0.08
*Casp1*	NM_012762	1.09 ± 0.11	1.04 ± 0.07	1.14 ± 0.64	1.08 ± 0.17
*Casp3*	NM_012922	1.18 ± 0.16	1.45 ± 0.23 *	1.25 ± 0.27	1.17 ± 0.02
*CD40*	NM_134360	1.00 ± 0.13	1.13 ± 0.33	0.83 ± 0.07	0.75 ± 0.08
*CD 40 lg*	NM_053353	1.56 ± 1.21	1.14 ± 0.43	1.71 ± 0.52	1.32 ± 0.21
*CXCL1*	NM_030845	2.99 ± 2.45	0.96 ± 0.47	3.93 ± 3.10	1.38 ± 0.77
*GSTA1*	NM_031509	0.86 ± 0.13	0.73 ± 0.03 *	0.67 ± 0.07	0.97 ± 0.05
*HMOX-1*	NM_012580	1.65 ± 0.33 *	1.31 ± 0.45	1.63 ± 0.81	1.27 ± 0.35
*Icam1*	NM_012967	1.09 ± 0.23	1.09 ± 0.08	0.79 ± 0.07 *	1.05 ± 0.09
*IL-1b*	NM_031512	1.12 ± 0.59	1.50 ± 0.27	1.36 ± 0.08	1.47 ± 0.14 *
*Il1r1*	NM_013123	1.01 ± 0.06	1.15 ± 0.09	0.90 ± 0.12	1.08 ± 0.06
*Il1rl1*	NM_013037	1.05 ± 0.48	1.12 ± 0.37	1.31 ± 0.55	1.21 ± 0.05 *
*Itga1*	NM_030994	1.44 ± 0.34	1.11 ± 0.02	1.05 ± 0.14	1.10 ± 0.08
*Itgam*	NM_012711	1.01 ± 0.22	1.10 ± 0.28	0.99 ± 0.26	1.34 ± 0.39
*Itgb1*	NM_017022	1.22 ± 0.48	1.09 ± 0.10	1.25 ± 0.90	1.08 ± 0.05
*Itgb2*	NM_001037780	1.59 ± 0.80	1.15 ± 0.06	1.17 ± 0.40	1.36 ± 0.04
*Mapk3*	NM_017347	1.13 ± 0.41	1.24 ± 0.19	0.80 ± 0.08	0.97 ± 0.11
*Mapk8*	NM_053829	1.60 ± 0.53	1.18 ± 0.13	0.82 ± 0.10	1.00 ± 0.16
*Mapk14*	NM_031020	1.20 ± 0.29	1.10 ± 0.03	0.91 ± 0.09	1.01 ± 0.08
*Nos2*	NM_012611	0.73 ± 0.47	1.03 ± 0.23	1.13 ± 0.75	0.28 ± 0.53
*NQO1*	NM_017000	1.16 ± 0.25	1.03 ± 0.13	1.11 ± 0.38	1.01 ± 0.12
*OGG1*	NM_030870	1.12 ± 0.19	1.16 ± 0.12	1.19 ± 0.10	1.10 ± 0.26
*Pde4d*	NM_001113328	1.14 ± 0.18	1.18 ± 0.07	1.09 ± 0.14	1.07 ± 0.04
*Pla2g7*	NM_001009353	1.13 ± 0.32	1.13 ± 0.55	1.72 ± 0.51	1.39 ± 0.11
*Plcb2*	NM_053478	1.30 ± 0.22	1.37 ± 0.25	0.83 ± 0.12	1.24 ± 0.13
*Plcb3*	NM_033350	1.11 ± 0.02	1.08 ± 0.03	1.02 ± 0.18	1.11 ± 0.14
*Plcd1*	NM_017035	1.39 ± 0.20 *	1.33 ± 0.13	0.97 ± 0.18	1.26 ± 0.20
*Ptgir*	NM_001077644	1.18 ± 0.50	1.23 ± 0.16	1.00 ± 0.18	1.03 ± 0.12
*Ptgis*	NM_031557	1.00 ± 0.14	1.06 ± 0.11	1.20 ± 0.46	0.85 ± 0.21
*Ptgs2*	NM_017232	1.39 ± 0.58	0.90 ± 0.37	1.76 ± 0.26	1.76 ± 1.50
*RPLPO*	NM_022402	0.85 ± 0.47	1.14 ± 0.08	0.50 ± 0.12	1.14 ± 0.04
*SOD2*	NM_017051	1.44 ± 0.33	0.96 ± 0.19	1.36 ± 0.20 *	1.14 ± 0.24
*TNF*	NM_012675	0.91 ± 0.57	1.14 ± 0.06	1.11 ± 0.47	1.31 ± 0.58
*Tnfrsf1a*	NM_013091	1.06 ± 0.24	1.07 ± 0.10	1.05 ± 0.21	0.99 ± 0.03
*Vcam1*	NM_012889	0.87 ± 0.23	1.33 ± 0.05 *	1.05 ± 0.13	1.20 ± 0.21

Rats were exposed by instillation to 0.36 mg of SiO_2__7 or SiO_2__40 and euthanized to isolate total RNA from the lungs either at 3 days (Exposure group; E) and 21 days (Recovery group; R) after initial instillation. Results obtained from TAC analysis represent the ratio of mRNA abundance as compared with respective control (unexposed) rats, normalized to the expression value of 18s RNA, ACTB, GAPDH, PPIA and calculated based on the comparative C_t_ (cycle threshold) method (2^−ΔΔCt^). * *p* < 0.05 versus control (Student’s *t*-test).

**Table 8:** Gene expression profile identified by TAC analysis following inhalation of SiO_2__7 in rats.

		SiO_2__7					
		STIS-E			STIS-R		
Gene Symbol	NM	0.5 mg/m^3^	2 mg/m^3^	5 mg/m^3^	0.5 mg/m^3^	2 mg/m^3^	5 mg/m^3^
*Adrb2*	NM_012492	0.68 ± 0.07 *	0.74 ± 0.01 *	0.60 ± 0.06 *	0.63 ± 0.02 *	0.56 ± 0.17 *	**0.65 ± 0.08**
*Anxa3*	NM_012823	0.70 ± 0.13	0.74 ± 0.10	0.49± 0.14 *	0.74 ± 0.08 *	0.62 ± 0.02 *	0.64 ± 0.04 *
*Casp1*	NM_012762	0.88 ± 0.06	0.72 ± 0.10	0.61 ± 0.07 *	1.05 ± 0.47	1.09 ± 0.22	1.02 ± 0.09
*Casp3*	NM_012922	0.96 ± 0.05	1.26 ± 0.22	0.83 ± 0.01	1.27 ± 0.40	0.82 ± 0.11 *	0.96 ± 0.07
*CD40*	NM_134360	3.33 ± 4.03	0.82 ± 0.37	1.04 ± 0.31	1.33 ± 0.46	1.03 ± 0.17	1.00 ± 0.17
*CD 40 lg*	NM_053353	1.21 ± 0.37	2.35 ± 0.75 *	1.05 ± 0.11	0.50 ± 0.05	0.48 ± 0.06	0.57 ± 0.06
*CXCL1*	NM_030845	2.34 ± 0.97	10.55 ± 8.86	7.25 ± 2.00 *	1.32 ± 0.25	1.53 ± 0.56	1.99 ± 1.12
*GSTA1*	NM_031509	0.47 ± 0.97	0.63 ± 0.31	0.48 ± 0.03	0.77 ± 0.08	0.93 ± 0.26	0.68 ± 0.14
*HMOX-1*	NM_012580	0.97 ± 0.20	**1.78 ± 0.36**	1.17 ± 0.05	0.86 ± 0.09	0.81 ± 0.17	0.89 ± 0.24
*Icam1*	NM_012967	1.04 ± 0.15	0.93 ± 0.13	0.86 ± 0.09	1.08 ± 0.08	0.93 ± 0.17	1.10 ± 0.04
*IL-1b*	NM_031512	2.05 ± 0.45 *	3.70 ± 0.39 *	2.91 ± 0.79 *	0.85 ± 0.02	0.49 ± 0.05 *	0.72 ± 0.13
*Il1r1*	NM_013123	0.96 ± 0.12	0.96 ± 0.13	0.89 ± 0.04 *	0.97 ± 0.08	0.72 ± 0.16 *	0.92 ± 0.07
*Il1rl1*	NM_013037	0.97 ± 0.28	1.19 ± 0.22	0.70 ± 0.05 *	0.94 ± 0.46	0.92 ± 0.21	0.93 ± 0.16
*Itga1*	NM_030994	1.16 ± 0.09	1.44 ± 0.42	0.96 ± 0.11	1.25 ± 0.16	0.84 ± 0.18	0.95 ± 0.10
*Itgam*	NM_012711	1.45 ± 0.42	3.10 ± 0.84 *	3.70 ± 1.18 *	1.01 ± 0.45	0.69 ± 0.27	1.00 ± 0.02
*Itgb1*	NM_017022	0.86 ± 0.15	0.74 ± 0.03 *	0.65 ± 0.05 *	0.71 ± 0.09 *	0.75 ± 0.16	0.81 ± 0.34
*Itgb2*	NM_001037780	0.90 ± 0.09	1.01 ± 0.19	0.83 ± 0.10	0.87 ± 0.07	0.76 ± 0.13 *	1.00 ± 0.11
*Mapk3*	NM_017347	0.90 ± 0.18	0.90 ± 0.12	0.85 ± 0.14	0.96 ± 0.29	0.85 ± 0.36	0.93 ±0.25
*Mapk8*	NM_053829	0.74 ± 0.23	0.98 ± 0.23	0.73 ± 0.07	0.87 ± 0.10	0.91 ± 0.33	1.33 ± 0.41
*Mapk14*	NM_031020	0.98 ± 0.09	1.01 ± 0.10	0.79 ± 0.07 *	1.08 ± 0.18	0.88 ± 0.08	0.96 ± 0.02
*Nos2*	NM_012611	5.13 ± 2.31 *	13.29 ± 3.29 *	11.71 ± 5.97 *	1.92 ± 2.45	0.46 ± 0.34	1.41 ± 1.39
*NQO1*	NM_017000	0.71 ± 0.05 *	0.96 ± 0.31	0.69 ± 0.05 *	1.31 ± 0.38	0.94 ± 0.22	1.10 ± 0.07
*OGG1*	NM_030870	0.79 ± 0.10	0.92 ± 0.18	0.59 ± 0.07	0.82 ± 0.10	0.93 ± 0.26	0.81 ± 0.07
*Pde4d*	NM_001113328	0.91 ± 0.27	1.04 ± 0.06	0.65 ± 0.10 *	1.02 ± 0.20	0.91 ± 0.21	1.03 ± 0.14
*Pla2g7*	NM_001009353	1.09 ± 0.23	2.10 ± 0.42 *	0.92 ± 0.24	0.77 ± 0.10	0.39 ± 0.06 *	0.83 ± 0.28
*Plcb2*	NM_053478	0.74 ± 0.08 *	1.31 ± 0.32	0.71 ± 0.11 *	1.04 ± 0.18	0.79 ± 0.14	0.99 ± 0.32
*Plcb3*	NM_033350	1.05 ± 0.16	1.18 ± 0.06 *	0.83 ± 0.04 *	0.99 ± 0.12	0.71 ± 0.10 *	0.81 ± 0.04 *
*Plcd1*	NM_017035	0.97 ± 0.20	1.09 ± 0.28	0.71 ± 0.06 *	0.68 ± 0.06	0.57 ± 0.12 *	0.60 ± 0.06 *
*Ptgir*	NM_001077644	0.84 ± 0.15	0.99 ± 0.26	0.70 ± 0.17	1.12 ± 0.12	0.89 ± 0.07	1.07 ± 0.35
*Ptgis*	NM_031557	0.83 ± 0.16	1.08 ± 0.29	0.68 ± 0.09 *	1.11 ± 0.23	1.00 ± 0.10	0.76 ± 0.05
*Ptgs2*	NM_017232	0.96 ± 0.30	2.31 ± 0.57 *	0.89 ± 0.39	1.55 ± 0.54	0.94 ± 0.59	0.77 ± 0.23
*RPLPO*	NM_022402	0.98 ± 0.02	1.57 ± 1.00	0.92 ± 0.06	1.39 ± 0.29	0.91 ± 0.18	0.96 ± 0.07
*SOD2*	NM_017051	1.19 ± 0.15	2.21 ± 0.40 *	2.68 ± 0.30 *	1.31 ± 0.68	1.11 ± 0.22	1.09 ± 0.11
*TNF*	NM_012675	2.05 ± 0.26 *	5.49 ± 1.90 *	6.39 ± 3.37 *	1.41 ± 0.34	1.28 ± 0.22	1.38 ± 0.53
*Tnfrsf1a*	NM_013091	0.90 ± 0.04	0.97 ± 0.21	0.80 ± 0.11	1.04 ± 0.13	0.85 ± 0.17	0.97 ± 0.06
*Vcam1*	NM_012889	0.95 ± 0.17	1.15 ± 0.10	1.07 ± 0.24	1.22 ± 0.11	1.32 ± 0.46	1.10 ± 0.24

Rats were exposed by inhalation to three doses of SiO_2__7 (0.5, 2 or 5 mg/m^3^), and rats were euthanized to isolate total RNA from the lungs directly after exposure (5 days, STIS-E) or after a recovery period of 21 days (STIS-R). Results obtained from TAC analysis represent the ratio of mRNA abundance as compared with respective control (unexposed) rats, normalized to the expression value of 18s RNA, ACTB, GAPDH, PPIA genes and calculated based on the comparative Ct (cycle threshold) method (2^−ΔΔCt^). * *p* < 0.05 versus control (Student’s *t*-test).

## Data Availability

The datasets used and/or analyzed during the current study are available from the corresponding authors on reasonable request.

## Supplementary Materials

The following are available online at https://www.mdpi.com/article/10.3390/nano11061502/s1, Table S1: Main physicochemical properties of the tested aSiO_2_ and TiO_2_ nanomaterials in deionized water, Table S2: Custom TaqMan Array Card (TAC) genes list.

## References

[1] Stark W.J., Stoessel P.R., Wohlleben W., Hafner A. (2015). Industrial applications of nanoparticles. Chem. Soc. Rev..

[2] Hyde E.D.E.R., Seyfaee A., Neville F., Moreno-Atanasio R. (2016). Colloidal Silica Particle Synthesis and Future Industrial Manufacturing Pathways: A Review. Ind. Eng. Chem. Res..

[3] Jeelani P.G., Mulay P., Venkat R., Ramalingam C. (2019). Multifaceted Application of Silica Nanoparticles. A Review. Silicon.

[4] Guichard Y., Maire M.-A., Sébillaud S., Fontana C., Langlais C., Micillino J.-C., Darne C., Roszak J., Stępnik M., Fessard V. (2015). Genotoxicity of synthetic amorphous silica nanoparticles in rats following short-term exposure, part 2: Intratracheal instillation and intravenous injection. Environ. Mol. Mutagen..

[5] Murugadoss S., Lison D., Godderis L., Brule S.V.D., Mast J., Brassinne F., Sebaihi N., Hoet P.H. (2017). Toxicology of silica nanoparticles: An update. Arch. Toxicol..

[6] IARC (2012). Arsenic, Metals, Fibres, and Dusts Volume 100 C A Review of Human Carcinogens.

[7] OECD (2016). Silicon Dioxide: Summary of the Dossier.

[8] ECETOC (2006). Synthetic Amorphous Silica.

[9] Gonzalez L., Lukamowicz-Rajska M., Thomassen L.C., Kirschhock C.E., Leyns L., Lison D., Martens J.A., Elhajouji A., Kirsch-Volders M. (2014). Co-assessment of cell cycle and micronucleus frequencies demonstrates the influence of serum on the in vitro genotoxic response to amorphous monodisperse silica nanoparticles of varying sizes. Nanotoxicology.

[10] Decan N., Wu D., Williams A., Bernatchez S., Johnston M., Hill M., Halappanavar S. (2016). Characterization of in vitro genotoxic, cytotoxic and transcriptomic responses following exposures to amorphous silica of different sizes. Mutat. Res. Genet. Toxicol. Environ. Mutagen..

[11] Guichard Y., Fontana C., Chavinier E., Terzetti F., Gaté L., Binet S., Darne C. (2016). Cytotoxic and genotoxic evaluation of different synthetic amorphous silica nanomaterials in the V79 cell line. Toxicol. Ind. Health.

[12] Haase A., Dommershausen N., Schulz M., Landsiedel R., Reichardt P., Krause B.-C., Tentschert J., Luch A. (2017). Genotoxicity testing of different surface-functionalized SiO2, ZrO2 and silver nanomaterials in 3D human bronchial models. Arch. Toxicol..

[13] Maser E., Schulz M., Sauer U.G., Wiemann M., Ma-Hock L., Wohlleben W., Hartwig A., Landsiedel R. (2015). In vitro and in vivo genotoxicity investigations of differently sized amorphous SiO2 nanomaterials. Mutat. Res. Genet. Toxicol. Environ. Mutagen..

[14] Pfuhler S., Downs T.R., Allemang A.J., Shan Y., Crosby M.E. (2017). Weak silica nanomaterial-induced genotoxicity can be explained by indirect DNA damage as shown by the OGG1-modified comet assay and genomic analysis. Mutagenesis.

[15] Chen M., von Mikecz A. (2005). Formation of nucleoplasmic protein aggregates impairs nuclear function in response to SiO_2_ nanoparticles. Exp. Cell Res..

[16] Li N., Ma L., Wang J., Zheng L., Liu J., Duan Y., Liu H., Zhao X., Wang S., Wang H. (2009). Interaction Between Nano-Anatase TiO2 and Liver DNA from Mice In Vivo. Nanoscale Res. Lett..

[17] Kim H.R., Kim M.J., Lee S.Y., Oh S.M., Chung K.H. (2011). Genotoxic effects of silver nanoparticles stimulated by oxidative stress in human normal bronchial epithelial (BEAS-2B) cells. Mutat. Res. Toxicol. Environ. Mutagen..

[18] Wang Z., Li N., Zhao J., White J.C., Qu P., Xing B. (2012). CuO Nanoparticle Interaction with Human Epithelial Cells: Cellular Uptake, Location, Export, and Genotoxicity. Chem. Res. Toxicol..

[19] Barnes C.A., Elsaesser A., Arkusz J., Smok A., Palus J., Leśniak A., Salvati A., Hanrahan J.P., de Jong W.H., Dziubałtowska E. (2008). Reproducible Comet Assay of Amorphous Silica Nanoparticles Detects No Genotoxicity. Nano Lett..

[20] Singh N., Manshian B., Jenkins G., Griffiths S.M., Williams P.M., Maffeis T.G., Wright C., Doak S.H. (2009). NanoGenotoxicology: The DNA damaging potential of engineered nanomaterials. Biomaterials.

[21] Doak S., Manshian B., Jenkins G., Singh N. (2012). In vitro genotoxicity testing strategy for nanomaterials and the adaptation of current OECD guidelines. Mutat. Res. Toxicol. Environ. Mutagen..

[22] Jugan M.-L., Barillet S., Simon-Deckers A., Herlin-Boime N., Sauvaigo S., Douki T., Carriere M. (2011). Titanium dioxide nanoparticles exhibit genotoxicity and impair DNA repair activity in A549 cells. Nanotoxicology.

[23] Magdolenova Z., Collins A., Kumar A., Dhawan A., Stone V., Dusinska M. (2014). Mechanisms of genotoxicity. A review of in vitro and in vivo studies with engineered nanoparticles. Nanotoxicology.

[24] Di Gioacchino M., Petrarca C., Lazzarin F., Di Giampaolo L., Sabbioni E., Boscolo P., Mariani-Costantini R., Bernardini G. (2011). Immunotoxicity of nanoparticles. Int. J. Immunopathol. Pharmacol..

[25] Dalle-Donne I., Aldini G., Carini M., Colombo R., Rossi R., Milzani A. (2006). Protein carbonylation, cellular dysfunction, and disease progression. J. Cell. Mol. Med..

[26] Davies M.J. (2005). The oxidative environment and protein damage. Biochim. Biophys. Acta Proteins Proteom..

[27] Nel A., Xia T., Mädler L., Li N. (2006). Toxic Potential of Materials at the Nanolevel. Science.

[28] Arts J.H., Muijser H., Duistermaat E., Junker K., Kuper C.F. (2007). Five-day inhalation toxicity study of three types of synthetic amorphous silicas in Wistar rats and post-exposure evaluations for up to 3months. Food Chem. Toxicol..

[29] Brown D.M., Kanase N., Gaiser B., Johnston H., Stone V. (2014). Inflammation and gene expression in the rat lung after instillation of silica nanoparticles: Effect of size, dispersion medium and particle surface charge. Toxicol. Lett..

[30] Landsiedel R., Ma-Hock L., Hofmann T., Wiemann M., Strauss V., Treumann S., Wohlleben W., Gröters S., Wiench K., Van Ravenzwaay B. (2014). Application of short-term inhalation studies to assess the inhalation toxicity of nanomaterials. Part. Fibre Toxicol..

[31] Morris A.S., Adamcakova-Dodd A., Lehman S.E., Wongrakpanich A., Thorne P.S., Larsen S.C., Salem A.K. (2016). Amine modification of nonporous silica nanoparticles reduces inflammatory response following intratracheal instillation in murine lungs. Toxicol. Lett..

[32] Yang M., Jing L., Wang J., Yu Y., Cao L., Zhang L., Zhou X., Sun Z. (2016). Macrophages participate in local and systemic inflammation induced by amorphous silica nanoparticles through intratracheal instillation. Int. J. Nanomed..

[33] Hong Y., Yun W.Q., Yue L.M., Shan L.C., Jian Z.J. (2017). Pulmonary Toxicity in Rats Caused by Exposure to Intratracheal Instillation of SiO2 Nanoparticles. Biomed. Environ. Sci..

[34] Sutunkova M.P., Solovyeva S.N., Katsnelson B.A., Gurvich V.B., Privalova L.I., Minigalieva I.A., Slyshkina T.V., Valamina I.E., Makeyev O.H., Shur V.Y. (2017). A paradoxical response of the rat organism to long-term inhalation of silica-containing submicron (predominantly nanoscale) particles of a collected industrial aerosol at realistic exposure levels. Toxicology.

[35] Großgarten M., Holzlechner M., Vennemann A., Balbekova A., Wieland K., Sperling M., Lendl B., Marchetti-Deschmann M., Karst U., Wiemann M. (2018). Phosphonate coating of SiO2 nanoparticles abrogates inflammatory effects and local changes of the lipid composition in the rat lung: A complementary bioimaging study. Part. Fibre Toxicol..

[36] Sayes C., Reed K.L., Glover K.P., Swain K.A., Ostraat M.L., Donner E.M., Warheit D.B. (2009). Changing the dose metric for inhalation toxicity studies: Short-term study in rats with engineered aerosolized amorphous silica nanoparticles. Inhal. Toxicol..

[37] Shin J.H., Jeon K., Kim J.K., Kim Y., Jo M.S., Lee J.S., Baek J.E., Park H.S., An H.J., Park J.D. (2017). Subacute inhalation toxicity study of synthetic amorphous silica nanoparticles in Sprague-Dawley rats. Inhal. Toxicol..

[38] Tarantini A., Huet S., Jarry G., Lanceleur R., Poul M., Tavares A.M., Vital N., Louro H., Silva M.J., Fessard V. (2015). Genotoxicity of synthetic amorphous silica nanoparticles in rats following short-term exposure. Part 1: Oral route. Environ. Mol. Mutagen..

[39] Nemmar A., Yuvaraju P., Beegam S., Pathan J., Kazzam E.E., Ali B.H. (2016). Oxidative stress, inflammation, and DNA damage in multiple organs of mice acutely exposed to amorphous silica nanoparticles. Int. J. Nanomed..

[40] Mu Q., Hondow N.S., Krzemiński Ł., Brown A.P., Jeuken L.J.C., Routledge M.N. (2012). Mechanism of cellular uptake of genotoxic silica nanoparticles. Part. Fibre Toxicol..

[41] OECD (2016). Test No. 489: In Vivo Mammalian Alkaline Comet Assay. OECD Guidelines for the Testing of Chemicals, Section 4.

[42] Doak S.H., Dusinska M. (2017). NanoGenotoxicology: Present and the future. Mutagenesis.

[43] Azqueta A., Dusinska M. (2015). The use of the comet assay for the evaluation of the genotoxicity of nanomaterials. Front. Genet..

[44] Collins A.R. (2014). Measuring oxidative damage to DNA and its repair with the comet assay. Biochim. Biophys. Acta Gen. Subj..

[45] Li Y., Yan J., Ding W., Chen Y., Pack L.M., Chen T. (2016). Genotoxicity and gene expression analyses of liver and lung tissues of mice treated with titanium dioxide nanoparticles. Mutagenesis.

[46] Asare N., Duale N., Slagsvold H.H., Lindeman B., Olsen A.K., Gromadzka-Ostrowska J., Meczynska-Wielgosz S., Kruszewski M., Brunborg G., Instanes C. (2015). Genotoxicity and gene expression modulation of silver and titanium dioxide nanoparticles in mice. Nanotoxicology.

[47] Gosens I., Costa P.M., Olsson M., Stone V., Costa A.L., Brunelli A., Badetti E., Bonetto A., Bokkers B.G., de Jong W.H. (2021). Pulmonary toxicity and gene expression changes after short-term inhalation exposure to surface-modified copper oxide nanoparticles. NanoImpact.

[48] Karkossa I., Bannuscher A., Hellack B., Bahl A., Buhs S., Nollau P., Luch A., Schubert K., Von Bergen M., Haase A. (2019). An in-depth multi-omics analysis in RLE-6TN rat alveolar epithelial cells allows for nanomaterial categorization. Part. Fibre Toxicol..

[49] Arts J.H., Irfan M.-A., Keene A.M., Kreiling R., Lyon D., Maier M., Michel K., Neubauer N., Petry T., Sauer U.G. (2016). Case studies putting the decision-making framework for the grouping and testing of nanomaterials (DF4nanoGrouping) into practice. Regul. Toxicol. Pharmacol..

[50] Bahl A., Hellack B., Balas M., Dinischiotu A., Wiemann M., Brinkmann J., Luch A., Renard B.Y., Haase A. (2019). Recursive feature elimination in random forest classification supports nanomaterial grouping. NanoImpact.

[51] Wiemann M., Vennemann A., Sauer U.G., Wiench K., Ma-Hock L., Landsiedel R. (2016). An in vitro alveolar macrophage assay for predicting the short-term inhalation toxicity of nanomaterials. J. Nanobiotechnol..

[52] Karkossa I., Bannuscher A., Hellack B., Wohlleben W., Laloy J., Stan M.S., Dinischiotu A., Wiemann M., Luch A., Haase A. (2020). Nanomaterials Induce Different Levels of Oxidative Stress, Depending on the Used Model System: Comparison of In Vitro and In Vivo Effects.

[53] Driessen M.D., Mues S., Vennemann A., Hellack B., Bannuscher A., Vimalakanthan V., Riebeling C., Ossig R., Wiemann M., Schnekenburger J. (2015). Proteomic analysis of protein carbonylation: A useful tool to unravel nanoparticle toxicity mechanisms. Part. Fibre Toxicol..

[54] Bannuscher A., Hellack B., Bahl A., Laloy J., Herman H., Stan M.S., Dinischiotu A., Giusti A., Krause B.-C., Tentschert J. (2020). Metabolomics profiling to investigate nanomaterial toxicity in vitro and in vivo. Nanotoxicology.

[55] NanoToxClass SOP Dispersion. https://www.nanopartikel.info/data/projekte/NanoToxClass/NanoToxClass-SOP_Dispersion_by_cup_horn_sonication_V2.0.pdf.

[56] Lozano O., Colaux J.L., Laloy J., Alpan L., Dogné J.-M., Lucas S. (2018). Fast, asymmetric and nonhomogeneous clearance of SiC nanoaerosol assessed by micro-particle-induced x-ray emission. Nanomedicine.

[57] Bessa M.J., Costa C., Reinosa J., Pereira C., Fraga S., Fernández J., Bañares M.A., Teixeira J.P. (2017). Moving into advanced nanomaterials. Toxicity of rutile TiO2 nanoparticles immobilized in nanokaolin nanocomposites on HepG2 cell line. Toxicol. Appl. Pharmacol..

[58] Møller P., Azqueta A., Boutet-Robinet E., Koppen G., Bonassi S., Milić M., Gajski G., Costa S., Teixeira J.P., Pereira C.C. (2020). Minimum Information for Reporting on the Comet Assay (MIRCA): Recommendations for describing comet assay procedures and results. Nat. Protoc..

[59] Bahl A., Hellack B., Wiemann M., Giusti A., Werle K., Haase A., Wohlleben W. (2020). Nanomaterial categorization by surface reactivity: A case study comparing 35 materials with four different test methods. NanoImpact.

[60] Ihaka R., Gentleman R. (1996). R: A Language for Data Analysis and Graphics. J. Comput. Graph. Stat..

[61] Carvalho B.S., Irizarry R.A. (2010). A framework for oligonucleotide microarray preprocessing. Bioinformatics.

[62] Gentleman R.C., Carey V.J., Bates D.M., Bolstad B., Dettling M., Dudoit S., Ellis B., Gautier L., Ge Y., Gentry J. (2004). Bioconductor: Open software development for computational biology and bioinformatics. Genome Biol..

[63] Schmittgen T.D., Livak K.J. (2008). Analyzing real-time PCR data by the comparative C T method. Nat. Protoc..

[64] Ashburner M., Ball C.A., Blake J., Botstein D., Butler H., Cherry J.M., Davis A.P., Dolinski K., Dwight S.S., Eppig J.T. (2000). Gene Ontology: Tool for the unification of biology. Nat. Genet..

[65] Yazdimamaghani M., Moos P.J., Dobrovolskaia M.A., Ghandehari H. (2019). Genotoxicity of amorphous silica nanoparticles: Status and prospects. Nanomed. Nanotechnol. Biol. Med..

[66] Neyrinck A., Van Hée V., Piront N., De Backer F., Toussaint O., Cani P.D., Delzenne N.M. (2012). Wheat-derived arabinoxylan oligosaccharides with prebiotic effect increase satietogenic gut peptides and reduce metabolic endotoxemia in diet-induced obese mice. Nutr. Diabetes.

[67] Osier M., Oberörster G. (1997). Intratracheal Inhalation vs Intratracheal Instillation: Differences in Particle Effects. Toxicol. Sci..

[68] ECETOC (2013). Poorly Soluble Particles/Lung Overload.

[69] Alessandrini F., Pimentel J.A.A., Landsiedel R., Wohlleben W., Mempel M., Marzaioli V., Weichenmeier I., Luxenhofer G., Wiemann M., Eiden S. (2014). Surface modifications of silica nanoparticles are crucial for their inert versus proinflammatory and immunomodulatory properties. Int. J. Nanomed..

[70] Gosens I., Post J.A., De La Fonteyne L.J.J., Jansen E.H.J.M., Geus J.W., Cassee F.R., De Jong W.H. (2010). Impact of agglomeration state of nano- and submicron sized gold particles on pulmonary inflammation. Part. Fibre Toxicol..

[71] Von Essen S.G., Robbins R.A., Thompson A.B., Ertl R.F., Linder J., Rennard S. (1988). Mechanisms of Neutrophil Recruitment to the Lung by Grain Dust Exposure. Am. Rev. Respir. Dis..

[72] Fujiwara N., Kobayashi K. (2005). Macrophages in inflammation. Curr. Drug Targets Inflamm. Allergy.

[73] Miyata R., van Eeden S.F. (2011). The innate and adaptive immune response induced by alveolar macrophages exposed to ambient particulate matter. Toxicol. Appl. Pharmacol..

[74] Liberman A.C., Budziñski M.L., Sokn C., Gobbini R.P., Steininger A., Arzt E. (2018). Regulatory and Mechanistic Actions of Glucocorticoids on T and Inflammatory Cells. Front. Endocrinol..

[75] Liberman A.C., Druker J., Perone M.J., Arzt E. (2007). Glucocorticoids in the regulation of transcription factors that control cytokine synthesis. Cytokine Growth Factor Rev..

[76] Abidin Z., Syafiq A., Rahim R.A., Arshad M., Khairuddin M., Nabilah F., Faudzi M., Voon C.H., Tang T.-H., Citartan M. (2017). Current and potential developments of cortisol aptasensing towards point-of-care diagnostics (POTC). Sensors.

[77] Lillehoj E.P., Kato K., Lu W., Kim K.C. (2013). Cellular and Molecular Biology of Airway Mucins. Int. Rev. Cell Mol. Biol..

[78] Davies D.E. (2009). The Role of the Epithelium in Airway Remodeling in Asthma. Proc. Am. Thorac. Soc..

[79] David R., Luu O., Damm E., Wen J., Nagel M., Winklbauer R. (2014). Tissue cohesion and the mechanics of cell rearrangement. Development.

[80] Tremi I., Havaki S., Georgitsopoulou S., Lagopati N., Georgakilas V., Gorgoulis V., Georgakilas A. (2021). A Guide for Using Transmission Electron Microscopy for Studying the Radiosensitizing Effects of Gold Nanoparticles In Vitro. Nanomaterials.

[81] Engin A.B., Nikitovic D., Neagu M., Henrich-Noack P., Docea A.O., Shtilman M.I., Golokhvast K., Tsatsakis A.M. (2017). Mechanistic understanding of nanoparticles’ interactions with extracellular matrix: The cell and immune system. Part. Fibre Toxicol..

[82] Huwyler J., Kettiger H., Schipanski A., Wick P. (2013). Engineered nanomaterial uptake and tissue distribution: From cell to organism. Int. J. Nanomed..

[83] Xu P., Van Kirk E.A., Zhan Y., Murdoch W.J., Radosz M., Shen Y. (2007). Targeted Charge-Reversal Nanoparticles for Nuclear Drug Delivery. Angew. Chem. Int. Ed..

[84] Asati A., Santra S., Kaittanis C., Perez J.M. (2010). Surface-Charge-Dependent Cell Localization and Cytotoxicity of Cerium Oxide Nanoparticles. ACS Nano.

[85] Li X., Tang Y., Xu L., Kong X., Zhang L., Chang Y., Zhao H., Zhang H., Liu X. (2017). Dependence between cytotoxicity and dynamic subcellular localization of up-conversion nanoparticles with different surface charges. RSC Adv..

[86] Soenen S.J., Rivera-Gil P., Montenegro J.-M., Parak W.J., De Smedt S.C., Braeckmans K. (2011). Cellular toxicity of inorganic nanoparticles: Common aspects and guidelines for improved nanotoxicity evaluation. Nano Today.

[87] Albi E. (2011). Role of intranuclear lipids in health and disease. Clin. Lipidol..

[88] Lehman S.E., Morris A.S., Mueller P.S., Salem A.K., Grassian V.H., Larsen S.C. (2016). Silica nanoparticle-generated ROS as a predictor of cellular toxicity: Mechanistic insights and safety by design. Environ. Sci. Nano.

[89] Wajner M., Amaral A.U. (2015). Mitochondrial dysfunction in fatty acid oxidation disorders: Insights from human and animal studies. Biosci. Rep..

[90] Rinaldo P., Cowan T.M., Matern D. (2008). Acylcarnitine profile analysis. Genet. Med..

[91] Pannkuk E.L., Laiakis E.C., Authier S., Wong K., Fornace A.J. (2016). Targeted metabolomics of nonhuman primate serum after exposure to ionizing radiation: Potential tools for high-throughput biodosimetry. RSC Adv..

[92] Ma-Hock L., Burkhardt S., Strauss V., Gamer A.O., Wiench K., Van Ravenzwaay B., Landsiedel R. (2009). Development of a Short-Term Inhalation Test in the Rat Using Nano-Titanium Dioxide as a Model Substance. Inhal. Toxicol..

[93] Klein C.L., Wiench K., Wiemann M., Ma-Hock L., Van Ravenzwaay B., Landsiedel R. (2012). Hazard identification of inhaled nanomaterials: Making use of short-term inhalation studies. Arch. Toxicol..

[94] Chen Z., Wang Y., Ba T., Li Y., Pu J., Chen T., Song Y., Gu Y., Qian Q., Yang J. (2014). Genotoxic evaluation of titanium dioxide nanoparticles in vivo and in vitro. Toxicol. Lett..

[95] Noël A., Truchon G. (2015). Inhaled Titanium Dioxide Nanoparticles: A Review of Their Pulmonary Responses with Particular Focus on the Agglomeration State. Nano Life.

[96] Naya M., Kobayashi N., Ema M., Kasamoto S., Fukumuro M., Takami S., Nakajima M., Hayashi M., Nakanishi J. (2012). In vivo genotoxicity study of titanium dioxide nanoparticles using comet assay following intratracheal instillation in rats. Regul. Toxicol. Pharmacol..

[97] Lindberg H.K., Falck G.C.-M., Catalán J., Koivisto A.J., Suhonen S., Järventaus H., Rossi E.M., Nykäsenoja H., Peltonen Y., Moreno C. (2012). Genotoxicity of inhaled nanosized TiO2 in mice. Mutat. Res. Toxicol. Environ. Mutagen..

[98] Relier C., Dubreuil M., Cordelli E., Mejia J., Eleuteri P., Robidel F., Loret T., Pacchierotti F., Lucas S., Lacroix G. (2017). Study of TiO 2 P25 nanoparticles genotoxicity on lung, blood and liver cells in lung overload and non-overload conditions after repeated respiratory exposure in rats. Toxicol. Sci..

[99] Jacobsen N.R., Møller P., Jensen K.A., Vogel U., Ladefoged O., Loft S., Wallin H. (2009). Lung inflammation and genotoxicity following pulmonary exposure to nanoparticles in ApoE-/-mice. Part. Fibre Toxicol..

[100] Kane A.B., Hurt R.H., Gao H. (2018). The asbestos-carbon nanotube analogy: An update. Toxicol. Appl. Pharmacol..

[101] Yuan X., Zhang X., Sun L., Wei Y., Wei X. (2019). Cellular Toxicity and Immunological Effects of Carbon-based Nanomaterials. Part. Fibre Toxicol..

