# Correction: Brandão et al. Genotoxicity and Gene Expression in the Rat Lung Tissue following Instillation and Inhalation of Different Variants of Amorphous Silica Nanomaterials (aSiO_2_ NM). *Nanomaterials* 2021, *11*, 1502

**DOI:** 10.3390/nano14231905

**Published:** 2024-11-27

**Authors:** Fátima Brandão, Carla Costa, Maria João Bessa, Elise Dumortier, Florence Debacq-Chainiaux, Roland Hubaux, Michel Salmon, Julie Laloy, Miruna S. Stan, Anca Hermenean, Sami Gharbia, Anca Dinischiotu, Anne Bannuscher, Bryan Hellack, Andrea Haase, Sónia Fraga, João Paulo Teixeira

**Affiliations:** 1 EPIUnit—Instituto de Saúde Pública, Universidade do Porto, Rua das Taipas, 4050-600 Porto, Portugal; fatimabrandao.988@gmail.com (F.B.); cstcosta@gmail.com (C.C.); mjbessa8@gmail.com (M.J.B.); jpft12@gmail.com (J.P.T.); 2 Laboratory for Integrative and Translational Research in Population Health (ITR), 4050-600 Porto, Portugal; 3 Environmental Health Department, National Institute of Health Dr. Ricardo Jorge, Rua Alexandre Herculano 321, 4000-053 Porto, Portugal; 4 ICBAS—Institute of Biomedical Sciences Abel Salazar, U. Porto—University of Porto, Rua de Jorge Viterbo Ferreira 228, 4050-313 Porto, Portugal; 5 Unité de Recherche en Biologie Cellulaire (URBC), Namur Research Institute for Life Sciences (Narilis), University of Namur, 5000 Namur, Belgium; elise.dumortier@gmail.com (E.D.); florence.chainiaux@unamur.be (F.D.-C.); 6 StratiCELL Laboratories, Research and Development, 5032 Les Isnes, Belgium; rhubaux@straticell.com (R.H.); msalmon@straticell.com (M.S.); 7 Namur Nanosafety Centre, Department of Pharmacy, Namur Research Institute for Life Sciences (Narilis), University of Namur, 5000 Namur, Belgium; julie.laloy@unamur.be; 8 Department of Biochemistry and Molecular Biology, University of Bucharest, 050095 Bucharest, Romania; miruna.stan@bio.unibuc.ro (M.S.S.); anca.hermenean@gmail.com (A.H.); samithgh2@hotmail.com (S.G.); anca.dinischiotu@bio.unibuc.ro (A.D.); 9 “Aurel Ardelean” Institute of Life Sciences, “Vasile Goldis” Western University of Arad, 310414 Arad, Romania; 10 Department of Chemical and Product Safety, German Federal Institute for Risk Assessment (BfR), 10589 Berlin, Germany; anne.bannuscher@unifr.ch (A.B.); Andrea.Haase@bfr.bund.de (A.H.); 11 Adolphe Merkle Institute (AMI), University of Fribourg, 1700 Fribourg, Switzerland; 12 Institute of Energy and Environmental Technology (IUTA) e.V., 47229 Duisburg, Germany; bryan.hellack@uba.de; 13 German Environment Agency (UBA), 06844 Dessau-Roβlau, Germany

In the original publication [1], there was a mistake in Table 5 as published. During the proofreading stage, Table 5 was accidentally duplicated with Table 6. The corrected Table 5 appears below.

The authors state that the scientific conclusions are unaffected. This correction was approved by the Academic Editor. The original publication has also been updated.

## Figures and Tables

**Table 5:** Main affected pathways in Gene Ontology (GO) in rats exposed by instillation to SiO_2__15_Unmod and SiO_2__15_Amino.

NM	Exposure Condition	Rank	Id	Size	*p* Value	Description
**SiO_2__15_Unmod**	**Exposure Group**	1	GO:0007127	83 (112)	6.00 × 10^−7^	meiosis I
		2	GO:0048247	42 (46)	3.50 × 10^−6^	lymphocyte chemotaxis
		3	GO:0071459	15 (21)	5.20 × 10^−6^	protein localization to chromosome, centromeric
		4	GO:0060340	10 (11)	8.20 × 10^−6^	positive regulation of type I interferon-mediated
		5	GO:0070098	63 (66)	1.30 × 10^−5^	chemokine-mediated signaling pathway
		6	GO:0072676	73 (78)	2.00 × 10^−5^	lymphocyte migration
		7	GO:0045132	67 (93)	3.20 × 10^−5^	meiotic chromosome segregation
		8	GO:0034501	13 (18)	4.20 × 10^−5^	protein localization to kinetochore
		9	GO:0034502	64 (76)	1.80 × 10^−4^	protein localization to chromosome
		10	GO:0070192	53 (72)	1.90 × 10^−4^	chromosome organization involved in meiotic cell cycle
	**Recovery Group**	1	GO:0048680	9 (11)	2.20 × 10^−5^	positive regulation of axon regeneration
		2	GO:0070572	10 (12)	8.80 × 10^−5^	positive regulation of neuron projection
		3	GO:2001223	10 (11)	1.60 × 10^−4^	negative regulation of neuron migration
		4	GO:2000849	11 (11)	1.60 × 10^−4^	regulation of glucocorticoid secretion
		5	GO:0071300	65 (76)	3.30 × 10^−4^	cellular response to retinoic acid
		6	GO:0003348	6 (6)	3.60 × 10^−4^	cardiac endothelial cell differentiation
		7	GO:0060956	6 (6)	3.60 × 10^−4^	endocardial cell differentiation
		8	GO:0006297	5 (5)	3.70 × 10^−4^	nucleotide-excision repair, DNA gap filling
		9	GO:0035933	12 (12)	5.10 × 10^−4^	glucocorticoid secretion
		10	GO:2000852	5 (5)	5.50 × 10^−4^	regulation of corticosterone secretion
**SiO_2__15_Amino**	**Exposure Group**	1	GO:0000910	96 (110)	1.50 × 10^−7^	cytokinesis
		2	GO:0000281	42 (47)	4.00 × 10^−7^	mitotic cytokinesis
		3	GO:0033260	23 (30)	5.40 × 10^−6^	nuclear DNA replication
		4	GO:0071459	15 (21)	7.60 × 10^−6^	protein localization to chromosome, centromeric
		5	GO:0051383	12 (14)	1.80 × 10^−5^	kinetochore organization
		6	GO:0051414	7 (7)	2.00 × 10^−5^	response to cortisol
		7	GO:0040020	31 (35)	2.30 × 10^−5^	regulation of meiotic nuclear division
		8	GO:0061640	49 (56)	2.50 × 10^−5^	cytoskeleton-dependent cytokinesis
		9	GO:0051445	45 (51)	2.50 × 10^−5^	regulation of meiotic cell cycle
		10	GO:1902969	9 (10)	3.90 × 10^−5^	mitotic DNA replication
	**Recovery Group**	1	GO:0035082	44 (60)	1.90 × 10^−14^	axoneme assembly
		2	GO:0003341	44 (64)	7.10 × 10^−13^	cilium movement
		3	GO:0001578	66 (89)	4.80 × 10^−8^	microtubule bundle formation
		4	GO:0002385	25 (39)	2.60 × 10^−5^	mucosal immune response
		5	GO:0060294	15 (17)	3.50 × 10^−5^	cilium movement involved in cell motility
		6	GO:0030199	38 (44)	5.10 × 10^−5^	collagen fibril organization
		7	GO:0001539	19 (22)	5.20 × 10^−5^	cilium or flagellum-dependent cell motility
		8	GO:0060285	19 (22)	5.20 × 10^−5^	cilium-dependent cell motility
		9	GO:0002251	26 (40)	5.20 × 10^−5^	organ or tissue specific immune response
		10	GO:0048755	13 (13)	6.50 × 10^−5^	branching morphogenesis of a nerve

Rats were exposed by instillation to 0.36 mg of SiO_2__15_Unmod or SiO_2__15_Amino and euthanized to isolate total RNA from the lungs both at 3 days (Exposure group; E) and 21 days (Recovery group; R) after initial instillation.
