# Supplementary material for: Genotoxicity and Gene Expression in the Rat Lung Tissue following Instillation and Inhalation of Different Variants of Amorphous Silica Nanomaterials (aSiO2 NM)
Source: Nanomaterials (Basel). 2021 Jun 7;11(6):1502. doi: 10.3390/nano11061502 (PMC8228975; doi:10.3390/nano11061502)
Supplement: Supplementary file 1 [file nanomaterials-11-01502-s001.zip › nanomaterials-1228953-supplementary.pdf]

nanomaterials

*Supplementary Materials*

# Genotoxicity and Gene Expression in the Rat Lung Tissue Following Instillation and Inhalation of Different Variants of Amorphous Silica Nanomaterials (a$SiO_2$ NM)

**Fátima Brandão [1,2,3,4], Carla Costa [1,2,3], Maria João Bessa [1,2,3,4], Elise Dumortier [5], Florence Debacq-Chainiaux [5], Roland Hubaux [6], Michel Salmon [6], Julie Laloy [7], Miruna S. Stan [8], Anca Hermenean [8,9], Sami Gharbia [8,9], Anca Dinischiotu [8], Anne Bannuscher [10,11], Bryan Hellack [12,13], Andrea Haase [10], Sónia Fraga [1,2,3,*] and João Paulo Teixeira [1,2,3]**

[1] EPIUnit—Instituto de Saúde Pública, Universidade do Porto, Rua das Taipas, 4050-600 Porto, Portugal; fatimabrandao.988@gmail.com (F.B.); cstcosta@gmail.com (C.C.); mjbessa8@gmail.com (M.J.B.); jpft12@gmail.com (J.P.T.)
[2] Laboratory for Integrative and Translational Research in Population Health (ITR), 4050-600 Porto, Portugal
[3] Environmental Health Department, National Institute of Health Dr. Ricardo Jorge, Rua Alexandre Herculano 321, 4000-053 Porto, Portugal
[4] ICBAS—Institute of Biomedical Sciences Abel Salazar, U. Porto—University of Porto, Rua de Jorge Viterbo Ferreira 228, 4050-313 Porto, Portugal
[5] Unité de Recherche en Biologie Cellulaire (URBC), Namur Research Institute for Life Sciences (Narilis), University of Namur, 5000 Namur, Belgium; elise.dumortier@gmail.com (E.D.); florence.chainiaux@unamur.be (F.D.-C.)
[6] StratiCELL Laboratories, Research and Development, 5032 Les Isnes, Belgium; rhubaux@straticell.com (R.H.); msalmon@straticell.com (M.S.)
[7] Department of Pharmacy, Namur Nanosafety Centre, Namur Research Institute for Life Sciences (Narilis), University of Namur, 5000 Namur, Belgium; julie.laloy@unamur.be (J.L.)
[8] Department of Biochemistry and Molecular Biology, University of Bucharest, 050095 Bucharest, Romania; miruna.stan@bio.unibuc.ro (M.S.S.); anca.hermenean@gmail.com (A.H.); samithgh2@hotmail.com (S.G.); anca.dinischiotu@bio.unibuc.ro (A.D.)
[9] "Aurel Ardelean" Institute of Life Sciences, "Vasile Goldis" Western University of Arad, 310414 Arad, Romania
[10] Department of Chemicals and Product Safety, German Federal Institute for Risk Assessment (BfR), 10589 Berlin, Germany; anne.bannuscher@unifr.ch (A.B.); Andrea.Haase@bfr.bund.de (A.H.)
[11] Adolphe Merkle Institute (AMI), University of Fribourg, 1700 Fribourg, Switzerland
[12] Institute of Energy and Environmental Technology (IUTA) e.V., 47229 Duisburg, Germany; bryan.hellack@uba.de
[13] German Environment Agency (UBA), 06844 Dessau-Roβlau, Germany
* Correspondence: sonia.fraga@insa.min-saude.pt; Tel.: +351-223-401143

**Table S1.** Main physicochemical properties of the tested a$SiO_2$ and $TiO_2$ nanomaterials in deionized water [1].

| NM property | $SiO_2$_7 | $SiO_2$_15_ Unmod | $SiO_2$_15_ Amino | $SiO_2$_40 | $TiO_2$_ NM-105 |
|---|---|---|---|---|---|
| Primary particle size (nm) | 8 [a] | 15 [a] | 15 [a] | 40 [a] | 21 [a] |
| Surface area ($m^2/g$) | 249 | 200[a] | 200[a] | 34 | 57 |
| Hydrodynamic diameter (nm) | 243 | 48 | 47 | 373 | 394 |
| Zeta potential pH 7.4 (mV) | −39.8 | −36.7 | −36.4 | −40.9 | −25.1 |
| Dissolution rate (% w) | 0.5 | 0.5 | 3.7 | 0.11 | 0.01 |
| Redox potential (mV) | 258 | 254 | 216 | 260 | 352 |
| EPR CPH (mass-based) | 0.93 | 0.82 | 0.92 | 0.68 | 0.69 |
| EPR CPH (surface-based) | 0.004 | 0.004 | 0.005 | 0.02 | 0.012 |
| EPR DMPO (mass-based) | 0.85 | 0.57 | 0.97 | 0.98 | 1.01 |
| EPR DMPO (surface-based) | 0.003 | 0.003 | 0.005 | 0.029 | 0.018 |

Primary particle size as provided by the manufacturer([a]); surface area as determined by Brunauer-Emmett-Teller (BET) and provided by the manufacturer; hydrodynamic diameter as determined by Dynamic Light Scattering (DLS); zeta potential as determined by Electrophoretic Light Scattering (ELS); dissolution rate in a steady-state system after 24 h shaking as determined by Inductively Coupled Plasma Optical Emission Spectrometry ICP-OES; redox potential measured using a potentiometer with platinum (Pt) electrode. Electron paramagnetic resonance (EPR) measurements using the spin probe CPH (EPR CPH) or DMPO (EPR CPH) were conducted - NM were applied at the same mass dose (mass-based) or at the same surface area dose (surface-based) and results were expresses in a sample to blank ratio.

**Table S2.** Custom TaqMan Array Card (TAC) genes list.

| Gene symbol | NM | Name |
|---|---|---|
| *18s* | NR_046237 | RNA, 18s ribosomal |
| *Actb* | NM_031144 | Actin beta |
| *Gapdh* | NM_017008 | Glyceraldehyde-3-phosphate dehydrogenase |
| *Ppia* | NM_017101 | Peptidylprolyl isomerase A (cyclophilin A) |
| *A2m* | NM_012488 | Alpha 2 macroglobulin |
| *Adrb2* | NM_012492 | Adrenergic receptor, beta 2 |
| *Anxa3* | NM_012823 | Annexin A3 |
| *Bdkrb1* | NM_030851 | Bradykinin receptor B1 |
| *Casp1* | NM_012762 | Caspase 1 |
| *Casp3* | NM_012922 | Caspase 3 |
| *CD40* | NM_134360 | CD40 |
| *CD 40 lg* | NM_053353 | CD 40 ligand |
| *CXCL1* | NM_030845 | C-X-C motif chemokine ligand 1 |
| *GSTA1* | NM_031509 | Glutathione S-transferase A1 |
| *HMOX-1* | NM_012580 | Heme Oxygenase-1 |
| *Hrh1* | NM_017018 | Histamine receptor H1 |
| *Hrh2* | NM_012965 | Histamine receptor H2 |
| *Icam1* | NM_012967 | Intercellular adhesion molecule 1 |
| *IFN-g* | NM_138880 | Interferon gamma |
| *IL-1b* | NM_031512 | Interleukin-1 beta |
| *Il1r1* | NM_013123 | Interleukin 1 receptor type 1 |
| *Il1rl1* | NM_013037 | Interleukin 1 receptor like 1 |
| *IL-6* | NM_012589 | Interleukin-6 |
| *IL-10* | NM_012854 | Interleukin-10 |
| *Itga1* | NM_030994 | Integrin subunit alpha L |
| *Itgam* | NM_012711 | Integrin subunit alpha M |
| *Itgb1* | NM_017022 | Integrin subunit beta 1 |
| *Itgb2* | NM_001037780 | Integrin subunit beta 2 |
| *Mapk3* | NM_017347 | Mitogen-activated protein kinase 3 |
| *Mapk8* | NM_053829 | Mitogen-activated protein kinase 8 |
| *Mapk14* | NM_031020 | Mitogen-activated protein kinase 14 |
| *Nos2* | NM_012611 | Nitric oxide synthase Inducible |
| *NQO1* | NM_017000 | NAD(P)H dehydrogenase quinone 1 |
| *OGG1* | NM_030870 | 8-oxoguanine DNA glycosylase |
| *Pde4d* | NM_001113328 | Phosphodiesterase 4B; cAMP specific |
| *Pla2g7* | NM_001009353 | Phospholipase A2; group VII (platelet-activating factor acetylhydrolase; plasma) |
| *Plcb2* | NM_053478 | Phospholipase C; beta 2 |
| *Plcb3* | NM_033350 | Phospholipase C; beta 3 |
| *Plcd1* | NM_017035 | Phospholipase C delta 1 |

| | | |
|---|---|---|
| *Ptgir* | NM_001077644 | Prostaglandin I2 receptor |
| *Ptgis* | NM_031557 | Prostaglandin I2 synthase |
| *Ptgs1* | NM_017043 | Prostaglandin-endoperoxide synthase 1 |
| *Ptgs2* | NM_017232 | Prostaglandin-endoperoxide synthase 2 |
| *RPLPO* | NM_022402 | Ribosomal protein lateral stalk subunit P0 |
| *SOD2* | NM_017051 | Superoxide Dismutase 2 |
| *TNF* | NM_012675 | Tumor Necrosis Factor-alpha |
| *Tnfrsf1a* | NM_013091 | TNF receptor superfamily member 1A |
| *Vcam1* | NM_012889 | Vascular Cell Adhesion Protein 1 |

## Reference

1. Bahl, A.; Hellack, B.; Balas, M.; Dinischiotu, A.; Wiemann, M.; Brinkmann, J.; Luch, A.; Renard, B.Y.; Haase, A. Recursive feature elimination in random forest classification supports nanomaterial grouping. *NanoImpact* **2019**, *15*, 100179.
